# Decadal variability in land carbon sink efficiency

**DOI:** 10.1186/s13021-021-00178-3

**Published:** 2021-05-10

**Authors:** Lei Zhu, Philippe Ciais, Ana Bastos, Ashley P. Ballantyne, Frederic Chevallier, Thomas Gasser, Masayuki Kondo, Julia Pongratz, Christian Rödenbeck, Wei Li

**Affiliations:** 1 Ministry of Education Key Laboratory for Earth System Modeling, Department of Earth System Science, Tsinghua University, Beijing, China; 2 Joint Center for Global Change Studies, Beijing, China; 3 Laboratoire des Sciences du Climat et de l’Environnement, LSCE/IPSL, CEA-CNRS-UVSQ, Université Paris-Saclay, Gif-sur-Yvette, France; 4 Department of Geography, Ludwig-Maximilians Universität, München, Germany; 5 Department of Biogeochemical Integration, Max Planck Institute for Biogeochemistry, Jena, Germany; 6 Department of Ecosystem and Conservation Sciences, WA Franke College of Forestry and Conservation, University of Montana, Missoula, MT USA; 7 International Institute for Applied Systems Analysis (IIASA), Laxenburg, Austria; 8 Institute for Space-Earth Environmental Research, Nagoya University, Nagoya, Japan; 9 Center for Global Environmental Research, National Institute for Environmental Studies, Tsukuba, Japan; 10 Max Planck Institute for Meteorology, Hamburg, Germany; 11 Department of Biogeochmical Systems, Max Planck Institute for Biogeochemistry, Jena, Germany

**Keywords:** Land carbon sink efficiency, Carbon neutrality, Trend reversal

## Abstract

**Background:**

The climate mitigation target of limiting the temperature increase below 2 °C above the pre-industrial levels requires the efforts from all countries. Tracking the trajectory of the land carbon sink efficiency is thus crucial to evaluate the nationally determined contributions (NDCs). Here, we define the instantaneous land sink efficiency as the ratio of natural land carbon sinks to emissions from fossil fuel and land-use and land-cover change with a value of 1 indicating carbon neutrality to track its temporal dynamics in the past decades.

**Results:**

Land sink efficiency has been decreasing during 1957–1990 because of the increased emissions from fossil fuel. After the effect of the Mt. Pinatubo eruption diminished (after 1994), the land sink efficiency firstly increased before 2009 and then began to decrease again after 2009. This reversal around 2009 is mostly attributed to changes in land sinks in tropical regions in response to climate variations.

**Conclusions:**

The decreasing trend of land sink efficiency in recent years reveals greater challenges in climate change mitigation, and that climate impacts on land carbon sinks must be accurately quantified to assess the effectiveness of regional scale climate mitigation policies.

**Supplementary Information:**

The online version contains supplementary material available at 10.1186/s13021-021-00178-3.

## Background

The Paris Agreement, aiming at limiting the temperature increase below 2 °C above pre-industrial levels, also aims at a greenhouse gas (GHG) balance between anthropogenic emissions and sinks in the second half of this century [1]. Although the Paris agreement focuses on anthropogenic fluxes, in reality it is hard to separate the anthropogenic contribution from global sinks [2]. Therefore, IPCC introduced the managed lands as a proxy for the place where anthropogenic emissions and removals occur [3]. But countries have a discretionary option to declare parts of their territory as being under management or not [4, 5], when evaluating or setting the mitigation targets e.g. in the national determined contributions (NDCs). Despite various possible interpretations of the exact GHG balance in the Paris Agreement [2], understanding trends and variations in the global balance between carbon sources and sinks is important for the evaluation of the NDCs to climate mitigation.

In the global carbon budget, the carbon source to the atmosphere includes emissions from fossil fuel (F) and land-use and land-cover change (L). These carbon emissions will be partly absorbed by land (‘natural’ carbon flux excluding land-use disturbed areas, B) and ocean (O), and the remaining part stays in the atmosphere. Enhanced B or O is thus needed to increase the carbon sink and to achieve the carbon neutrality. During the past 10 years (2009–2018), the land sink (B) has removed 33% of the anthropogenic emissions (F + L), and many future climate mitigation options rely on terrestrial ecosystems, such as afforestation, bioenergy with carbon capture and storage and enhanced soil carbon sequestration [4, 6–8]. The land sink thus plays a key role in meeting the target of carbon neutrality.

To integrate the information of anthropogenic emissions and land sinks, the instantaneous land carbon sink efficiency (E) is defined here as the ratio of B to the sum of F and L, i.e. E = B/(F + L). A higher value of E means more carbon emission absorbed by land, contributing to a slower growth rate of atmospheric CO_2_ concentration. E = 1 indicates a carbon neutral region where natural sinks fully offset land use emissions and fossil fuel emissions (a positive sign of carbon fluxes being adopted for carbon emissions to the atmosphere for F and L, and for carbon uptake from the atmosphere for B). E integrates trends from both emissions and sinks and thus is relevant for assessing regional trajectories with respect to carbon neutrality. The concept of E is the same as the fraction of the total emissions (F + L) absorbed by land [9]. However, previous studies did not report the trend of E in the past decade and analyze the regional E, which can be used as an integrated measure of the carbon neutrality accounting for both territorial emissions and sinks [5].

Changes in individual carbon fluxes contribute to the variations of E. For example, global annual F emissions have been increasing from an average of 3.04 ± 0.41 Pg C year^−1^ in the 1960s to 9.5 ± 0.4 Pg C year^−1^ in the past decade (2009−2018), largely driven by an increase in China [6, 10]. Global annual L emissions ranged from 1.0 to 1.8 Pg C year^−1^ since 1959, mainly contributed by tropical regions (South and Southeast Asia, Latin America and Sub-Saharan Africa) [6, 11]. However, emissions from L that are already reported under the United Nations Framework Convention on Climate Change (UNFCCC) are generally much lower than L based on the scientific definitions of Global Carbon Project (GCP), because they incompletely account for land degradation emissions, do not account for changes in cropland and grassland management intensity, ignore some conversions of carbon rich biomes like tropical forests becoming plantations (e.g. oil palm, rubber). B increased from 1.86 ± 0.53 Pg C year^−1^ in 1980–1984 to 2.82 ± 0.50 Pg C year^−1^ in 2010–2014 [12].

The components of the land sink efficiency (i.e. F, L and B) are influenced by both anthropogenic activities and natural factors. F is directly contributed by fossil fuel emissions caused by anthropogenic activities, while L could be increased by deforestation which results in carbon emissions. Secondary regrowth of forests (e.g., afforestation and reforestation) has the potential to reduce L [13–15]. The rising atmospheric CO_2_ concentrations can enhance plant photosynthesis (“CO_2_ fertilization”) and thus may increase B [15–18], although the CO_2_ response of carbon sequestration in mature forests may be insignificant [19]. Volcanic eruption and large-scale fire events are also important components that regulate B, both locally and globally [20–24]. Climate conditions like temperature and precipitation have multiple effects on the land carbon uptake. Although global warming has extended the growing season length and thus enhanced vegetation productivity in the northern temperate and boreal regions [25], this may be offset by autumn warming having led to carbon losses from northern ecosystems due to respiration increase [26]. ENSO-induced temperature and precipitation variations also strongly impact the carbon cycles in the tropics and play a dominant role in the variability of land sinks [27].

The objective of this study is to characterize the global and regional trajectories of land carbon sink efficiencies over the past decades. Considering the differences among various estimates of carbon fluxes, we use multiple datasets (mostly from the global carbon budget [6]) to validate our findings. We firstly analyze the trend of E on the global scale and identify critical regions that dominate it. We further analyze the trend of each individual carbon flux and the potential driving factors. Finally, we do a set of sensitivity tests (e.g., using multiple data sources) and discuss the implication of the land sink efficiency. Our analyses focus on the period covered by atmospheric inversion estimates, especially after 1990s when expansion of the atmospheric station network allowed for latitudinal resolution of surface fluxes.

## Methods

### Net land sink and fossil fuel emissions from atmospheric Inversion

We used annual net land sink (BL = B-L) and F data from two atmospheric inversions: the Copernicus Atmosphere Monitoring Service inversion (CAMS) [28] and the Jena CarboScope inversion (available at http://www.bgc-jena.mpg.de/CarboScope/) [29, 30]. CarboScope inversions combine fixed fossil fuel emission and ocean flux priors, and adjust land flux with its prescribed uncertainties to match atmospheric CO_2_ observations, while CAMS inversion has fixed fossil fuel emission priors and adjusts land and ocean fluxes. Fluxes and atmospheric CO_2_ mole fractions are linked to each other by a transport model. Although F is fixed, BL is estimated based on the atmospheric data, including the spatial distribution of carbon sinks across different regions.

The temporal coverage and spatial resolution of CAMS (v18.3) are 1979–2018 and 1.875° latitude × 3.75° longitude. The number of stations used in the CAMS increased over time as they became available, and a total of 129 stations were used in 2018. From the Jena CarboScope inversion (v4.3) we analyze five products using different station networks, including a network of stations having measurements from at least 1976 (hereafter Jena_s76, 9 stations), from 1981 (Jena_s81, 14 stations), from 1985 (Jena_s85, 21 stations) or from 1993 (Jena_s93, 35 stations), respectively. Moreover, we used the Jena CarboScope run sEXTocNEET_v4.3 (hereafter Jena_sNEET) using a growing network of 89 stations starting from 1957, but with year-independent degrees of freedom regressing interannual BL variations against variations in air temperature. The spatial resolution is 3.75° latitude × 5° longitude. Because the prior fixed fossil fuel emissions (F) are different in these two inversions (Additional file 1: Figure S1b), directly impacting posterior BL estimates, the BL data from the Jena inversions were adjusted to a common F value, as done in Peylin et al. [31] and Thompson et al. [32]. For global F, we used the values from global carbon budget [33], and for regional distribution of F we used the values from CAMS. All bunker fuels are considered as a surface fossil CO_2_ source distributed proportionally to national emissions shares of the global total [10, 33].

We also compare the results from the inversions with estimates of BL at the scale of both hemispheres over 1994–2013, using a two-box inversion and data from the two longest CO_2_ monitoring stations from South Pole and Mauna Loa, with ocean sinks from an ensemble of ocean biogeochemical models [34].

### Natural land sink as residual of GCP's annual global carbon budget

We calculated annual global B over 1959–2018 from the global carbon budget [32] as a residual of F, L from bookkeeping models, global atmospheric CO_2_ growth, and ocean sinks from an ensemble of ocean biogeochemical models as well. Deriving B as a residual term has recently been replaced by explicit simulations with dynamic global vegetation models (DGVMs) in recent versions of the annual global carbon budget [35]. Nevertheless, here we stay with the residual approach because the land sink from DGVMs added to ocean sinks and F emissions do not match the CO_2_ growth rate, with an imbalance ranging from − 1.75 to 1.96 PgC year^−1^ during 1959–2018, so that B from this approach is not consistent with atmospheric data [33, 36]. We considered B from the TRENDY-V8 DGVMs used in Friedlingstein et al [33] as a sensitivity test, considering that potential error terms in the other fluxes will be attributed to B with residual approach.

### Land-use and land-cover change flux from bookkeeping models and DGVMs

We mainly used L from the Bookkeeping of Land Use Emissions model [BLUE, 37] because this dataset is grid-based and updated to the latest year. The BLUE model used gridded LUC data from the Land Use Harmonization dataset [38]. In addition, we used L from three other sources as sensitivity tests: (1) L from the bookkeeping model by Houghton and Nassikas [10, hereafter H&N], (2) L from DGVMs in Friedlingstein et al [33], and (3) L from the OSCAR compact Earth system model that emulates the carbon cycle of TRENDY-V7 DGVMs [OSCAR, 39]. L from H&N is estimated based on country-level response curves of carbon pools for different LUC types and FAO/FRA forest data [11]. It should be noted that although H&N and BLUE are used as equally likely in the GCP, H&N is not used here for the prime analysis due to its ending in 2015 and lack of spatially explicit values after that year (GCP extended the results of H&N to 2018 only on global scale). In the GCP, DGVMs performed two simulations using different settings: S2 with varying CO_2_ and climate but time-invariant preindustrial land use maps, and S3 with annually updated CO_2_, climate and land use maps. L is thus the net biome productivity (NBP) difference between S2 and S3, which includes a foregone land sink in S2, leading to a component of L known as “loss of additional sink capacity” (LASC) which does not exist in observation-based estimates and in bookkeeping models [40, 41]. OSCAR embeds processes and parameters calibrated using outputs from DGVMs and calculates L using a bookkeeping method [39]. While L from DGVMs include LASC, L from OSCAR does not and can be compared with other bookkeeping models based on observations of carbon densities [39].

All carbon flux datasets used in this study are summarized in Additional file 1: Table S1.

### Other data

Atmospheric CO_2_ concentrations [42], temperature and precipitation from CRUJRA2.0 [43, 44], Multivariate ENSO Index (MEI) [45] and the Pacific Decadal Oscillation index (PDO) [46] were used to analyze the influencing factors of land sink efficiency. MEI and PDO index are widely used to describe the varying ocean and atmosphere conditions. We also used forest area gain derived from ESA CCI (European Space Agency Climate Change Initiative) yearly land cover maps from 1993–2018 to investigate the legacy land sink from forest gain.

### Data analysis

Gridded datasets with different spatial resolutions were resampled to 1° × 1°. We only focused on the period covered by inversion data (1957–2018) and used annual mean values of the carbon fluxes and climate variables. Because of the strong interannual variability (Additional file 1: Figure S1), we applied a 5-year moving window to carbon fluxes to better detect trends. In the atmospheric inversion output, however, it is impossible to distinguish B and L separately because the inversions do not include an explicit representation of L. We thus used L simulated by BLUE to calculate B. We also calculated trends of each individual flux on the regional scale for 11 regions (see region division in Additional file 1: Figure S2) to elucidate which region dominates the trend of E. Piecewise regression (“Segmented” package in R) was applied on global and regional trends to detect breakpoints. In addition, a linear least-square regression was used to calculate the trends during 1957–1990 and the trends before and after a detected breakpoint for each individual flux.

Because the global trend in B/(F + L) is not equal to the sum of regional trends, we used a method of removing-one-region at a time to analyze the regional contributions to the breakpoint of global land sink efficiency. Specifically, assume there is a breakpoint detected in the global signal, and the slopes before and after the breakpoint are s_1_ and s_2_. After removing B, F and L fluxes from one region, we assume that B/(F + L) from the sum of the other regions still shows a similar breakpoint, but the slopes before and after the breakpoint change to s_1_′ and s_2_′. We define the contribution of a region as a ratio of slope change (R_sc_):

$${\mathrm{R}}_{\mathrm{sc}}=1- \frac{{\mathrm{s}}_{2}^{\mathrm{^{\prime}}}-{\mathrm{s}}_{1}^{\mathrm{^{\prime}}}}{{\mathrm{s}}_{2}-{\mathrm{s}}_{1}} \times 100\mathrm{\%}$$

A positive value of R_sc_ indicates that this region strengthens the slope reversal, i.e., making the contribution to the global breakpoint more significant. Conversely, a negative value indicates the region weakens the slope reversal.

## Results

### Global trends

**Fig. 1:**
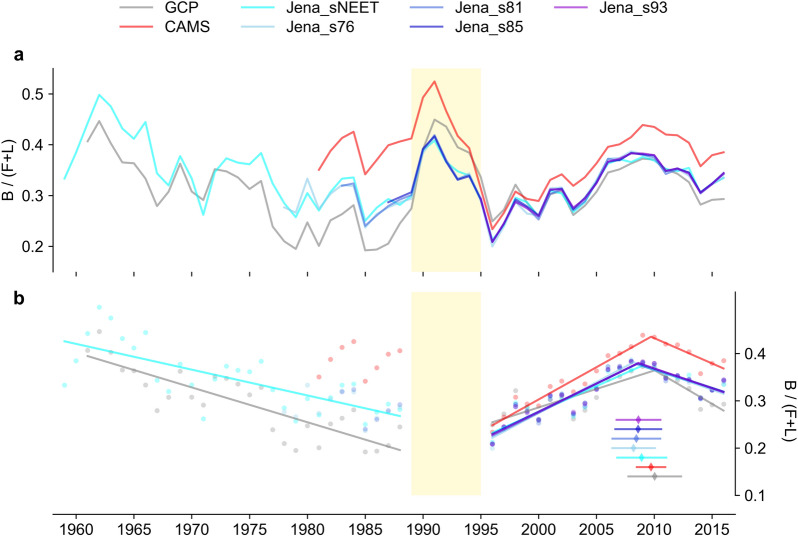
Land sink efficiencies based on 5-yr moving averages from different datasets during 1959–2016: **a** annual values, **b** annual values (dot) with linear regression (line) from 1959 to 1988 and piecewise regression (line) from 1996–2016. B in the inversion datasets (CAMS and Jena) is calculated by BL−L where L from BLUE is used. The diamonds and error bars in (b) indicate the detected breakpoints with 95% confidence interval. The Pinatubo eruption period is marked in light yellow shade. Note that we used 5-yr moving average fluxes, and thus the analysis period is shown as 1959–2016 instead of 1957–2018 (the original annual values)

Global land sink efficiencies (E) calculated from different datasets during 1957–2018 with 5-year moving average are shown in Fig. 1a. Note that the analysis period is shown as 1959–2016 because of the 5-year moving average. Higher E values are found during 1991–1993 because of the Mt. Pinatubo eruption that enhanced the land sink and the value of E peaked at around 0.5. Before this exceptional period, E shows a monotonically decreasing trend from the residual sink of GCP (1961–1988, p < 0.01) and from Jena_sNEET (1959–1988, p < 0.01, Fig. 1b). The results from CAMS (1981–1988), Jena_s76 (1978–1988) and Jena_s81 (1983–1988) start too late to detect a significant trend before the Mt. Pinatobo period. It is also possible that the decreasing trend was weakened or disappeared in the 1980s. The decreasing trend during 1959–1988 is mainly caused by the increasing trend of F during this period considering little changes in B and smaller absolute values of L and its trend (Additional file 1: Figure S1). After the Mt. Pinatubo eruption period, E increases until around 2009 and then decreases afterward (i.e., the trend reversal, Fig. 1b). This trend is present in all considered datasets, and the detected breakpoints range from 2008 to 2010 (p < 0.01). In the following, we focus mainly on E after the eruption of Mt. Pinatubo (i.e., from 1994–2018) because more datasets with better observation constraints are available during this period.

The original flux B and F are generally increasing during 1957–2018 (Additional file 1: Figure S1). B from different datasets is roughly consistent, but L from different dataset shows large variations over 1957–2018 owing to the various methods and input datasets (Additional file 1: Figure S1) [39].

### Regional contributions

**Fig. 2:**
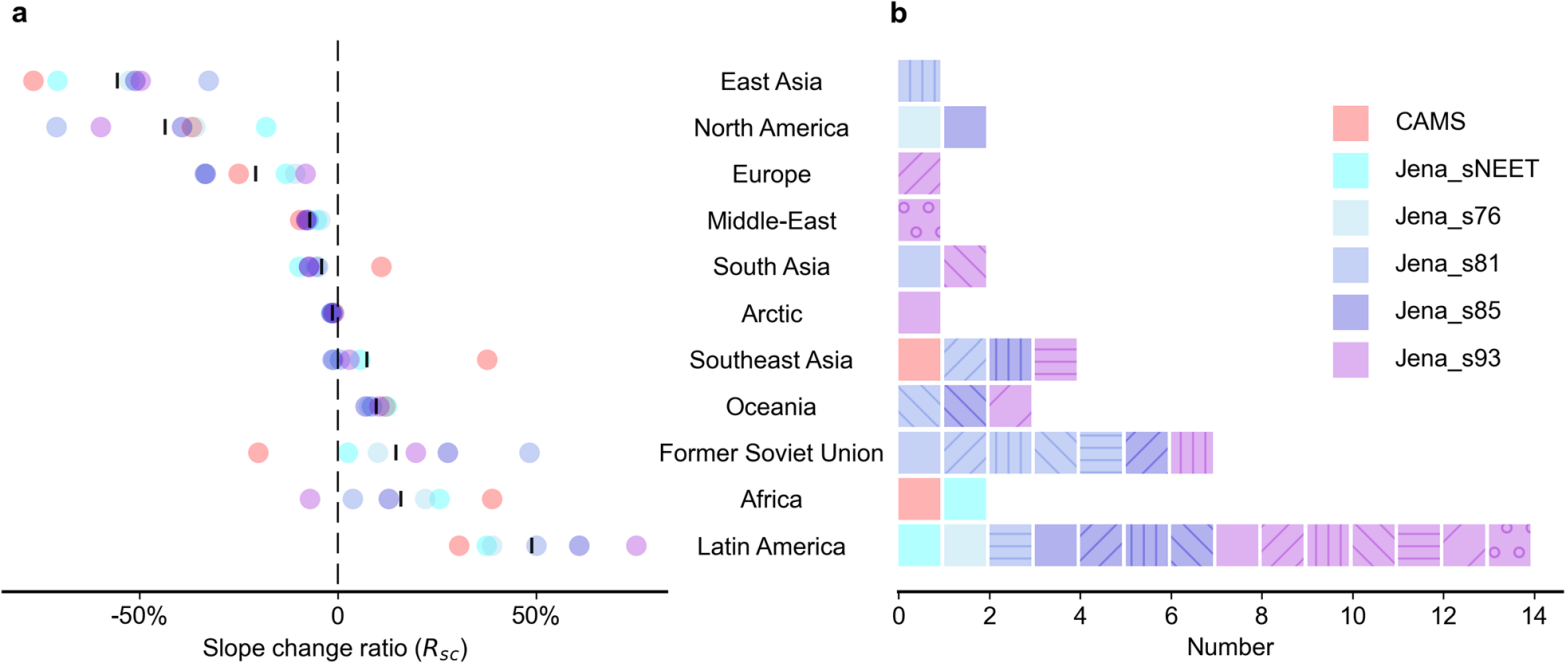
Regional contributions to the trend reversal after 2009. **a** Slope change ratio in different regions from various datasets. The short vertical black line is the mean value of the 6 datasets (colored dots). A positive (negative) value of R_sc_ indicates that this region strengthens (weakens) the trend reversal after the breakpoint. **b** Removal of pairs of regions that leads to the insignificance (p > 0.1) or disappearance of the trend reversal. Squares with the same hatch are one removal of 2-region combination

In general, the global breakpoint around 2009 remains detectable after removing contributions from any one individual region in both inversions and is all significant (p < 0.1) except when removing Latin America in Jena_s93 (Additional file 1: Figure S3–S8). The regional contributions could be clearly reflected by their R_sc_ values (Fig. 2a). The mean values of R_sc_ show that Latin America contributes most to the trend reversal in the Jena inversions with the largest positive R_sc_ values (i.e., enhancing effect on the trend reversal) while East Asia shows largest opposite effects with the most negative R_sc_ values. The negative contributions from North America, Europe and Middle-East are also consistent in all datasets (R_sc_ < 0 for all). However, Former Soviet Union shows more positive contributions than Africa in Jena_s81, Jena_s85 and Jena_s93 (Fig. 2a) but opposite contributions are found in CAMS (Fig. 2a). East Asia, North America and Europe all have high F and the most negative R_sc_ values, which proved that the trend reversal was not due to a change of F in any of these large F emitting regions.

Given the robustness of the global breakpoint after removing each individual region (Additional file 1: Figure S3–S8), we further removed 2 regions each time to examine whether the breakpoint would disappear. We find that several 2-regions combinations can make the breakpoint insignificant (p > 0.1) or disappear (Fig. 2b). Among these combinations, Latin America is the most frequently identified region in the Jena inversions and Former Soviet Union ranks second. However, these two regions are not sensitive regions in CAMS. In CAMS, only removing the combination of Africa and Southeast Asia can change the breakpoint. After removing any combination without Latin America, Africa, Former Soviet Union and Southeast Asia, the breakpoint still exists, confirming the minor or even opposite contributions of these four regions.

Although the two-box inversion cannot detect the breakpoint due to the short time series after 2009, it confirms that the increasing trend before 2009 is contributed by the Southern Hemisphere (Additional file 1: Figure S9).

### Individual fluxes in each region

**Fig. 3:**
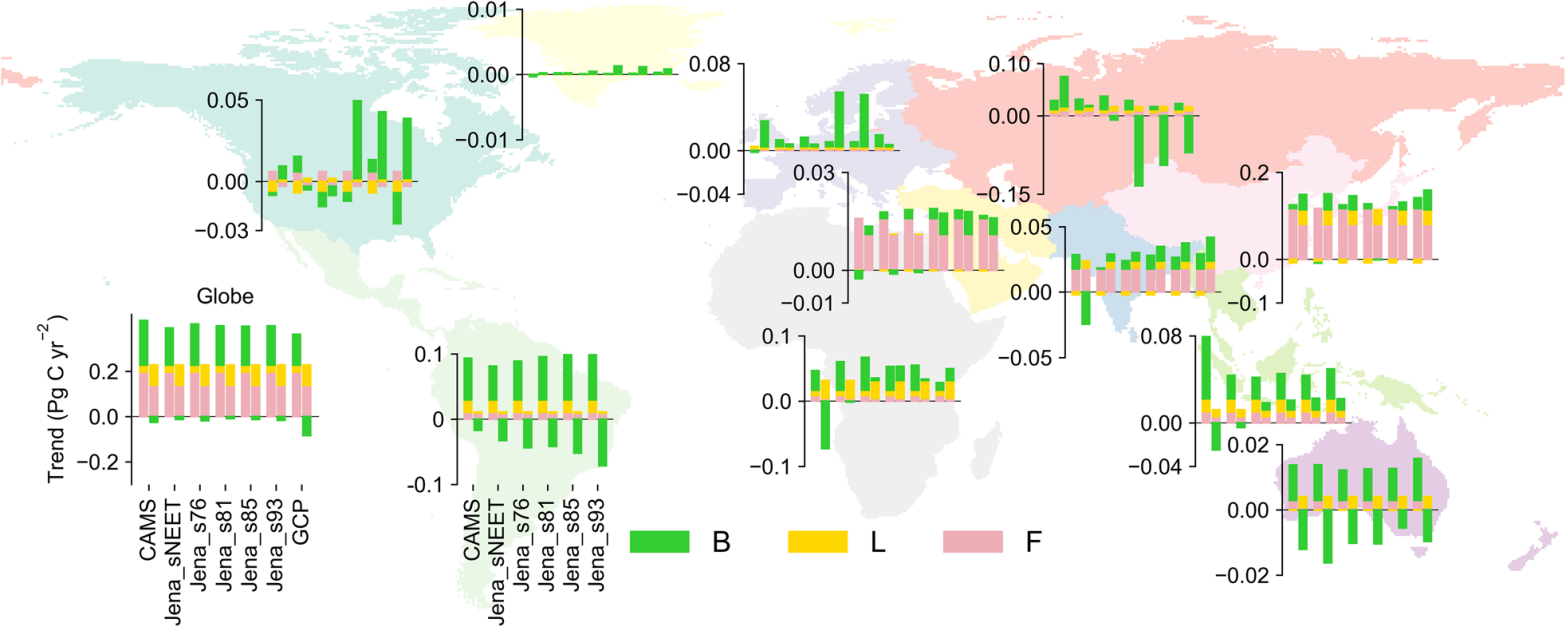
Global and regional trends in carbon fluxes before and after 2009 from 1996 to 2016 based on 5-year moving average. The left and right stacks of bars in each dataset represent trends before and after 2009, respectively. Note that data from GCP are only shown on the global scale. Categorical variables on the x-axis are consistent among bar plots for each region, with reference being given for Latin America. Y axes have different scales in different regions for legibility

Because it is partly ambiguous to simply decompose the trends in global E = B/(F + L) into trends in regional E, we calculate the global and regional trends in each individual flux before and after 2009 from 1996 to 2016 in different datasets (Fig. 3). Globally, F and L are increasing before and after 2009 while B is increasing only before 2009. After 2009, the growing trend of F weakens while the growing trend of L strengthened, albeit with considerable variability among estimates (Additional file 1: Figure S1c). The trend in B changes from positive to negative after 2009. Therefore, the reversal of trends in E are driven predominantly by the trend in B and, to a lesser extent, by the trend in L. The trend in F, on the other hand, shows a more slowly increasing rate after 2009 than before 2009, making the E value more positive after 2009. Thus, the trend in E is primarily driven by land sink variability (i.e., B), and F emissions have an opposite effect but with small magnitude.

The global trend in each carbon flux is the sum of regional trends. The global trend in F is mostly driven by trends in East Asia where the growth of fossil fuel emissions slows down after 2009 compared to that before 2009 (Fig. 3). A slow-down in F growth also appears in Africa, Middle East, Oceania and Southeast Asia with a smaller magnitude. In North America, however, F shows a small magnitude of decreasing trend after 2009.

Trends in B vary across different datasets in most regions (Fig. 3). In Africa, Southeast Asia and South Asia, B is increasing before 2009 but decreasing after 2009 in CAMS, which is not evident in most Jena datasets. However, the increasing trends in B are smaller in the Jena datasets in Africa and Southeast Asia after 2009. In the Former Soviet Union, Europe and North America, trends in B even show large variations across different datasets. A strong decreasing trend in B in the Former Soviet Union is found in Jena_s81, Jena_s85 and Jena_s93 after 2009.

In Latin America and Oceania, however, all datasets show a high consistency (Fig. 3). The sign of trends in B is positive before 2009 and negative after 2009. Although the trends in B are robust in Oceania, the values of the trends are very small and thus have limited contributions to the global trends. On the contrary, the strong reversed trends in B in Latin America largely explain the reversed trends in global B.

The increased positive trends in global L after 2009 are mainly contributed by Africa and East Asia. North America, South Asia and Oceania also contribute to these increasing positive trends but with a small magnitude. However, the large increasing trend of L in Latin America slows down after 2009 with stabilizing deforestation rates at low levels (Additional file 1: Figure S10, [47]).

## Discussion

We found a decreasing trend of E from 1957–1990 which is in line with the previous finding that the proportion of carbon emissions remaining in the atmosphere (airborne fraction) was increasing during this period [9]. After the Mt. Pinatubo eruption period (after 1994), E increased again and then decreased after 2009. To verify the robustness of this breakpoint, we did the following sensitivity tests: (1) using L from other three data sources (H&N, DGVMs and OSCAR), B from DGVMs and B from the residual of different ocean flux estimates; (2) removing adjustment of fossil fuel emissions on Jena data; (3) applying different moving average methods; and ((4) masking the latest El Niño event.

In general, our breakpoint is robust regardless of different choices of datasets and methods. Although there are large differences in L among different datasets (Additional file 1: Figure S1c), the absolute values of L are relatively small compared with F and B, and the breakpoint detection in E is robust regardless of different L choices (p < 0.01, Additional file 1: Figure S11). This breakpoint is, however, not reflected using B from DGVMs, mainly due to the non-significant increase of B before 2009 (Additional file 1: Figure S12). DGVMs likely underestimated the increase of B in the 2000s [34]. On the other hand, the decreasing trend of E after 2009 in DGVMs (Additional file 1: Figure S12) is consistent with the trend detected by inversion data. A previous study also found that the global difference in land sink between DGVMs and inversion datasets agreed well with the budget imbalance in the global carbon budget [36], indicating a possible bias in the land sink simulated by DGVMs. We also tested the ocean sink from each individual ocean model estimate and pCO_2_-based product of the global ocean sink reported in the global carbon budget, instead of the ensemble mean, to calculate the residual B in the global carbon budget, and the breakpoint of global E still exists (p < 0.01, Additional file 1: Figure S13). Without the adjustment of fossil fuel emissions on the Jena data (see "Net land sink and fossil fuel emissions from atmospheric Inversion" section), the global pattern remains consistent (p < 0.01, Additional file 1: Figure S14). To evaluate the influence of moving average methods, we applied 3-year moving average on the time series, and significant global breakpoint around 2009 in E is found in each dataset (p < 0.1, Additional file 1: Table S2). Even using the original annual values without moving averages, the breakpoint around 2009 is still detectable although not significant (Additional file 1: Table S2). After replacing the B in the strong El Niño years (2015 and 2016) with the averaged B of 2014 and 2017, the breakpoint detection is still significant after 5-yr moving average in CAMS and three Jena datasets (p < 0.1, Additional file 1: Table S2), indicating that this reversal is not completely caused by the latest strong El Niño event during 2015–2016.

To estimate the uncertainty of E, we estimated the uncertainty of each carbon flux after Mt. Pinatubo eruption, which is our focused period. Following GCP, F has a fixed uncertainty of 5% for all years, mainly contributed by the amounts of fuel consumed, the carbon and heat contents of fuels, and the combustion efficiency [6, 48]. The uncertainties of L and BL were estimated by calculating the standard deviation of all available fluxes (4 L estimates and 7 BL estimates in Additional file 1: Table S1) respectively. Finally, we used a Monte Carlo method to estimate the uncertainty of E. Specifically, we randomly selected F, BL and L from their distributions for 10,000 times and calculated the corresponding E. The standard deviation of E was taken as the uncertainty range (Additional file 1: Figure S15). We found that the uncertainty range of E is smaller than the interannual variability, indicating that the trend reversal can be robustly detected given the uncertainty in E (Additional file 1: Table S3, Figure S15).

These sensitivity tests and uncertainty analysis further confirm the robustness of the breakpoint and support that the smaller increasing trend of B is the dominant factor determining the reversed trend in land sink efficiency after 2009.

Compared to the period before 2009, the acceleration of the L growth in Africa and East Asia together with the weakening or relatively stable trend in B in the tropics, especially in Latin America, result in the weakening of E after 2009 (Fig. 3). This is consistent with the dominant role of tropical regions in the interannual variability of the global carbon cycle [49–52].

Climate variations play an important role in the reversed trend in land sink efficiency after 2009. In fact, 5-yr moving averages of MEI and PDO index both show a strong increasing trend since 2009 (Additional file 1: Figure S16a), which influences the land carbon sink. However, the impacts of ENSO and PDO on E seem to be small (Fig. 1) during the increasing phase of ENSO and PDO in the previous cycle (2000–2004), probably because of reduced intensity and shorter duration (Additional file 1: Figure S16b). In fact, if we apply multiple linear regression using annual precipitation, temperature and CO_2_ concentration to predict B in tropics and use the predicted B to calculate tropical E, the same breakpoint in the predicted E trend around 2009 is still significant (Additional file 1: Figure S17a), indicating that these factors can largely explain the trend reversal. Note that MEI is strongly correlated with temperature and precipitation in the tropics (Additional file 1: Figure S17b). It is well known that climate variations caused by e.g. El Niño may influence the tropical carbon cycle through different processes in different regions [27].

Climate impacts on the land sink may need to be accurately quantified in the future. Extreme El Niño events that strongly reduce tropical land carbon sinks are expected to be more frequent due to future greenhouse warming [53], but the trend of El Niño/La Niña intensity still remains unclear. While El Niño/La Niña cycles affect E, El Niño impacts are probably compensated by recovery fluxes in the subsequent years [54]. If they can fully offset each other, ENSO impacts on E may be negligible in the long term. On the other hand, if there is an anthropogenic fingerprint in trends in El Niño/La Niña intensity [55], climate change impact on the natural land sink needs to be considered to define mitigation goals compatible with the Paris agreement, for example, using a buffer pool approach [56]. In addition, PDO and other low frequency variability patterns might affect climate at the time scales these mitigation policies should be acting (the next few decades). They could either result in additional CO_2_ in the atmosphere (e.g. by imposing more drought/higher temperatures) amplifying the impacts or reduce it temporarily (if they would lead to some cooling and wetter conditions). Their impacts on the natural sink thus need to be accurately quantified to avoid a false sense of implementation progress or failure when assessing the collective result of climate mitigation policies. Currently, there is not enough evidence to identify the most likely of these two possibilities. Nevertheless, it is necessary to track the efficiency of natural sink dynamics.

Saturation of land carbon sinks could also contribute to the reversal of trends in land sink efficiency after 2009. Processes that regulate the land sink via atmospheric composition change (e.g. CO_2_ fertilization, nitrogen deposition), climate change (e.g. rising temperature) and LUC (e.g. forest regrowth, woody encroachment) are unlikely to be sustained permanently. Land carbon sinks are thus likely to decrease as the terrestrial carbon storage saturates. The saturation of the tropical land sink is already indicated by a network of Amazonian forest plots [52] and modelling studies [57]. However, some of this potential saturation may be offset by secondary forest regrowth in the tropics [13, 58]. Forest area gain in the tropics during 2009–2013 is lower than over the period before 2009 but became higher in the recent period after 2013 (Additional file 1: Figure S18). Linking forest area gain to the net land sink, however, remains challenging due to the uncertainties in the forest gain detection from remote sensing, biomass growth and soil carbon dynamics. Therefore, the contribution of legacy land sink from forest gain needs to be further investigated with emerging evidence.

Some countries have recently made commitments to achieve carbon neutrality in the future. For example, European Union proposed the ‘European Green Deal’ in 2019 which aimed at net zero emissions of GHGs by 2050 [59], and China also promised to be carbon neutrality before 2060 in the 75th session of the United Nations General Assembly. However, the decreasing land sink efficiency in recent years may make these ambitious aims more challenging. In future, we should keep tracing the trend of land sink efficiency and call for more efforts to progress towards the global carbon neutrality objective.

## Conclusions

Our study defined the land sink efficiency and explored its trends from 1957 to 2018 using multiple datasets. We found that before the Pinatubo eruption, the land sink efficiency was generally decreasing due to the increase in fossil fuel emissions. After the Mt. Pinatubo eruption, a trend reversal around 2009 was observed because of the changed land sink in tropical regions. Our results highlight the importance of the variations of the natural land sink, which are not the focus of the Paris agreement. The decreasing trend of land sink efficiency in the recent years reveals greater challenges in the mitigation of climate change and reinforces the need for more efforts to reduce anthropogenic emissions from fossil fuel burning and LUC to achieve the ambitious goals of the Paris Agreement.

## Supplementary Information

**Additional file 1.** Additional figures and tables.

## Data Availability

Global Carbon Budget data used in this study can be accessed from https://doi.org/10.18160/gcp-2019 [33]. Jena CarboScope inversion data is available at http://www.bgc-jena.mpg.de/CarboScope/. Copernicus Atmosphere Monitoring Service (CAMS) inversion data is available at https://ads.atmosphere.copernicus.eu/cdsapp#!/dataset/cams-global-greenhouse-gas-inversion?tab=overview. Atmospheric CO_2_ concentrations is available at https://www.esrl.noaa.gov/gmd/ccgg/trends/data.html. Temperature and precipitation data from CRUJRA2.0 are available at https://catalogue.ceda.ac.uk/uuid/7f785c0e80aa4df2b39d068ce7351bbb. Multivariate ENSO Index is available at https://www.esrl.noaa.gov/psd/enso/mei/. Pacific Decadal Oscillation index is available at https://www.ncdc.noaa.gov/teleconnections/pdo/. ESA CCI yearly land cover maps from 1993 to 2018 is available at http://maps.elie.ucl.ac.be/CCI/viewer/download.php.

## Abbreviations

GHG: Greenhouse gas
NDCs: National determined contributions
E: Instantaneous land carbon sink efficiency
B: ‘Natural’ carbon flux excluding land-use disturbed areas
F: Emissions from fossil fuel
L: Emissions from land-use and land-cover change
UNFCCC: United Nations Framework Convention on Climate Change
GCP: Global Carbon Project
LUC: Land-use change
ENSO: El-Niño-Southern Oscillation
CAMS: Copernicus Atmosphere Monitoring Service
R_sc_: Slope change values
H&N: Houghton and Nassikas
DGVMs: Dynamic global vegetation models
MEI: Multivariate ENSO Index
PDO: Pacific Decadal Oscillation
ESA CCI: European Space Agency Climate Change Initiative
