# Supplementary material for: Decadal variability in land carbon sink efficiency
Source: Carbon Balance Manag. 2021 May 10;16:15. doi: 10.1186/s13021-021-00178-3 (PMC8112069; doi:10.1186/s13021-021-00178-3)
Supplement: Supplementary file 1 — Additional file 1. Additional figures and tables. [file 13021_2021_178_MOESM1_ESM.docx]

Additional file 1:

**Decadal variability in land carbon sink efficiency reveals apparent trend reversal after 2009**

**Lei Zhu^1,2^, Philippe Ciais^3^, Ana Bastos^4,5^, Ashley P. Ballantyne^3,6^, Frederic Chevallier^3^, Thomas Gasser^7^, Masayuki Kondo^8,9^, Julia Pongratz^4,10^, Christian Rödenbeck^11^, Wei Li^1,2^***

^1^Ministry of Education Key Laboratory for Earth System Modeling, Department of Earth System Science, Tsinghua University, Beijing, China

^2^Joint Center for Global Change Studies, Beijing, China

^3^Laboratoire des Sciences du Climat et de l’Environnement, LSCE/IPSL, CEA-CNRS-UVSQ, Université Paris-Saclay, Gif-sur-Yvette, France

^4^Department of Geography, Ludwig-Maximilians Universität, München, Germany

^5^Department of Biogeochemical Integration, Max Planck Institute for Biogeochemistry, Jena, Germany

^6^Department of Ecosystem and Conservation Sciences, WA Franke College of Forestry and Conservation, University of Montana, Missoula, MT, USA

^7^International Institute for Applied Systems Analysis (IIASA), Laxenburg, Austria

^8^Institute for Space-Earth Environmental Research, Nagoya University, Nagoya, Nagoya, Aichi Japan

^9^Center for Global Environmental Research, National Institute for Environmental Studies, Tsukuba, Japan

^10^Max Planck Institute for Meteorology, Hamburg, Germany

^11^Department of Biogeochmical Systems, Max Planck Institute for Biogeochemistry, Jena, Germany

Corresponding author: Wei Li (wli2019@tsinghua.edu.cn)

**Contents of this file**

Figures S1 to S18

Tables S1 to S3


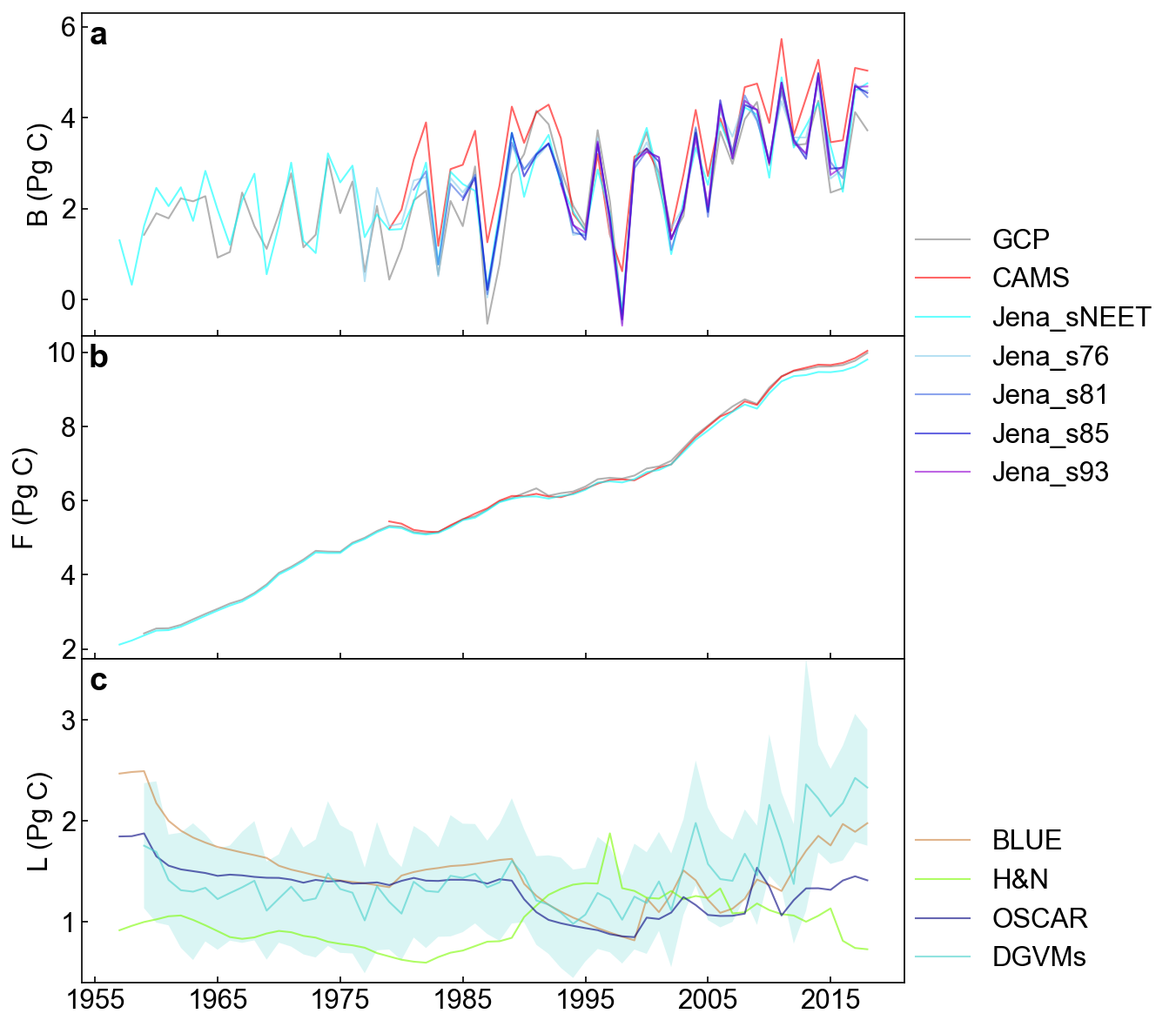


**Figure S1.** Annual carbon fluxes in different datasets. (a) Natural land sink (B). B in the inversion datasets is calculated by BL−L where L from BLUE is used. (b) Fossil fuel emission (F). (c) Emission from land-use change (L). The sky-blue shade is 1-σ uncertainties for DGVMs.


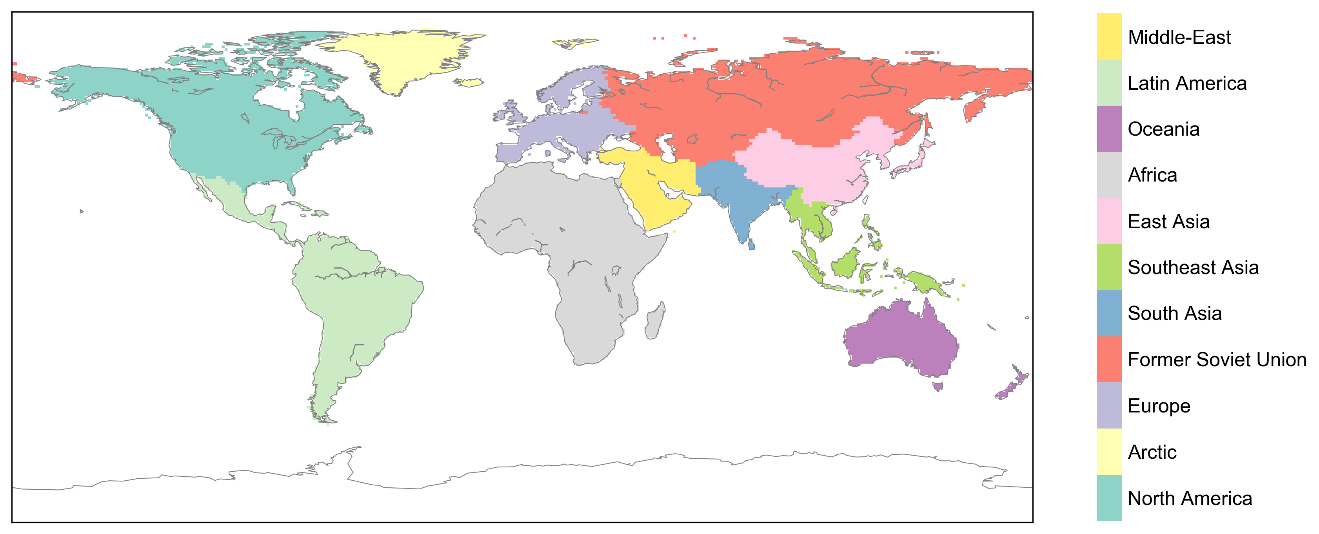


**Figure S2.** Division of the land surface into the 11 regions used in this study.


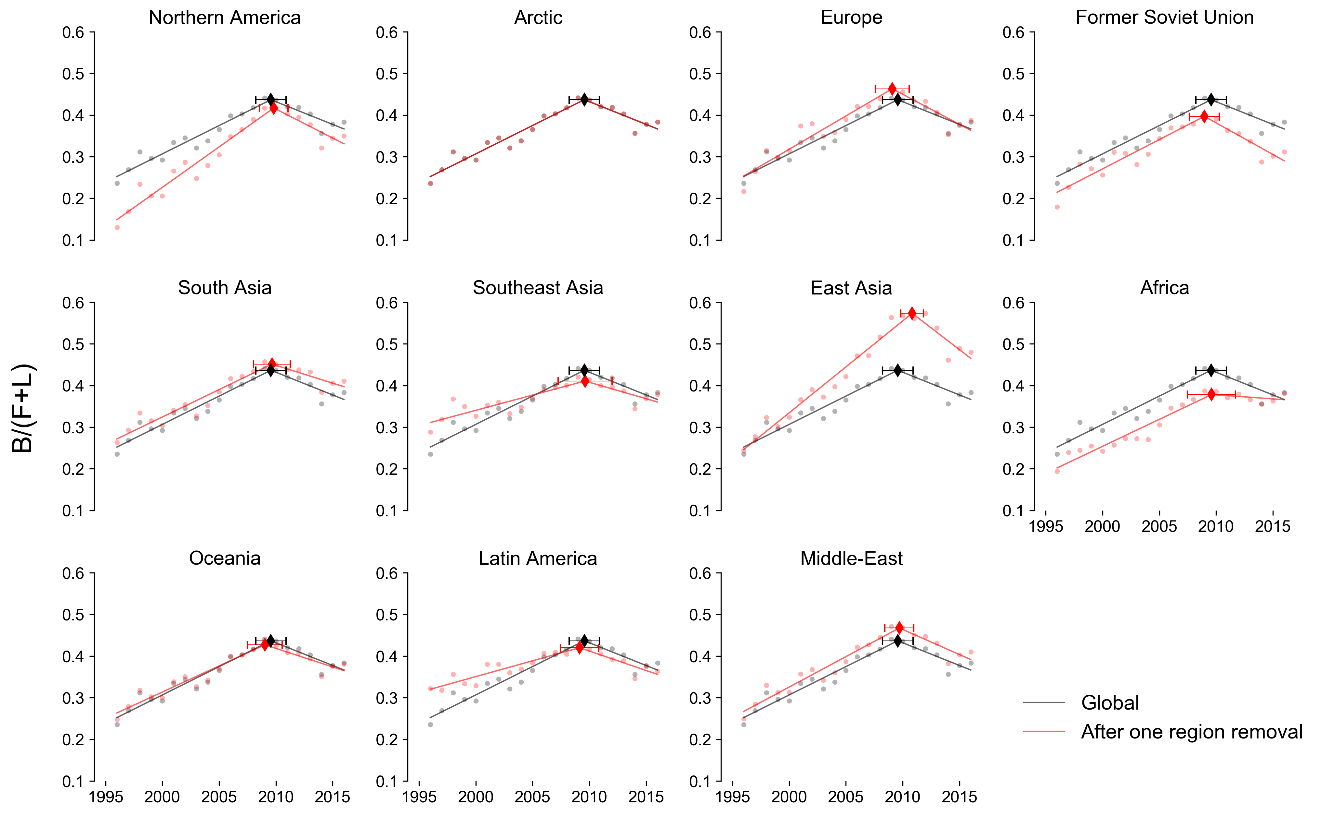


**Figure S3.** Global breakpoint pattern after removing fluxes from one individual region in CAMS from 1996-2016. Dots and lines represent the annual 5-yr moving average values and the piecewise regression lines, respectively. The black ones are for the global values and thus same for all subplots. The red ones represent the values calculated after removing the B and (F+L) fluxes in this region. The diamonds and error bars indicate the detected breakpoints with 95% confidence interval. Note that we used 5-yr moving average fluxes, and thus the analysis period is shown as 1996-2016 instead of 1994-2018 (the original annual values).


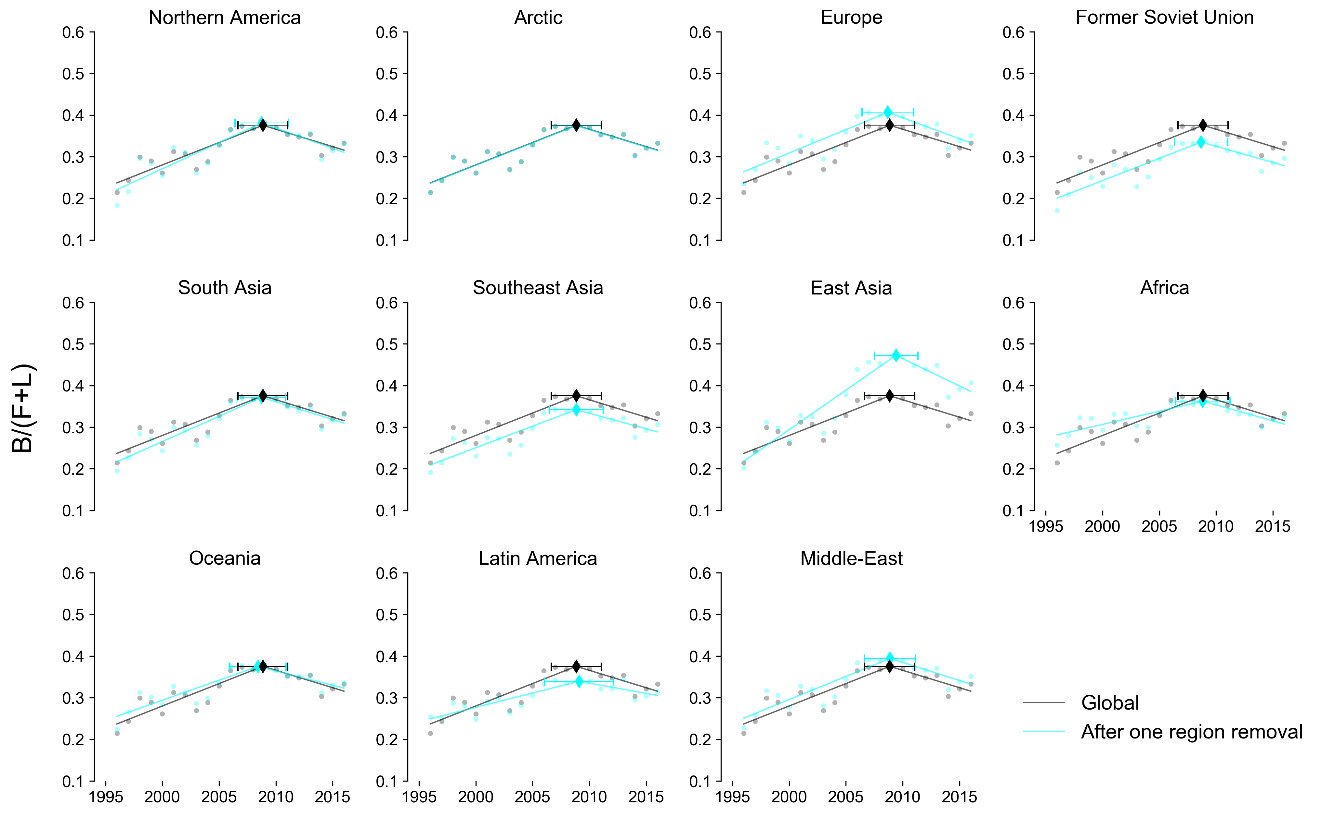


**Figure S4.** Same as Figure S3 but for Jena_sNEET.


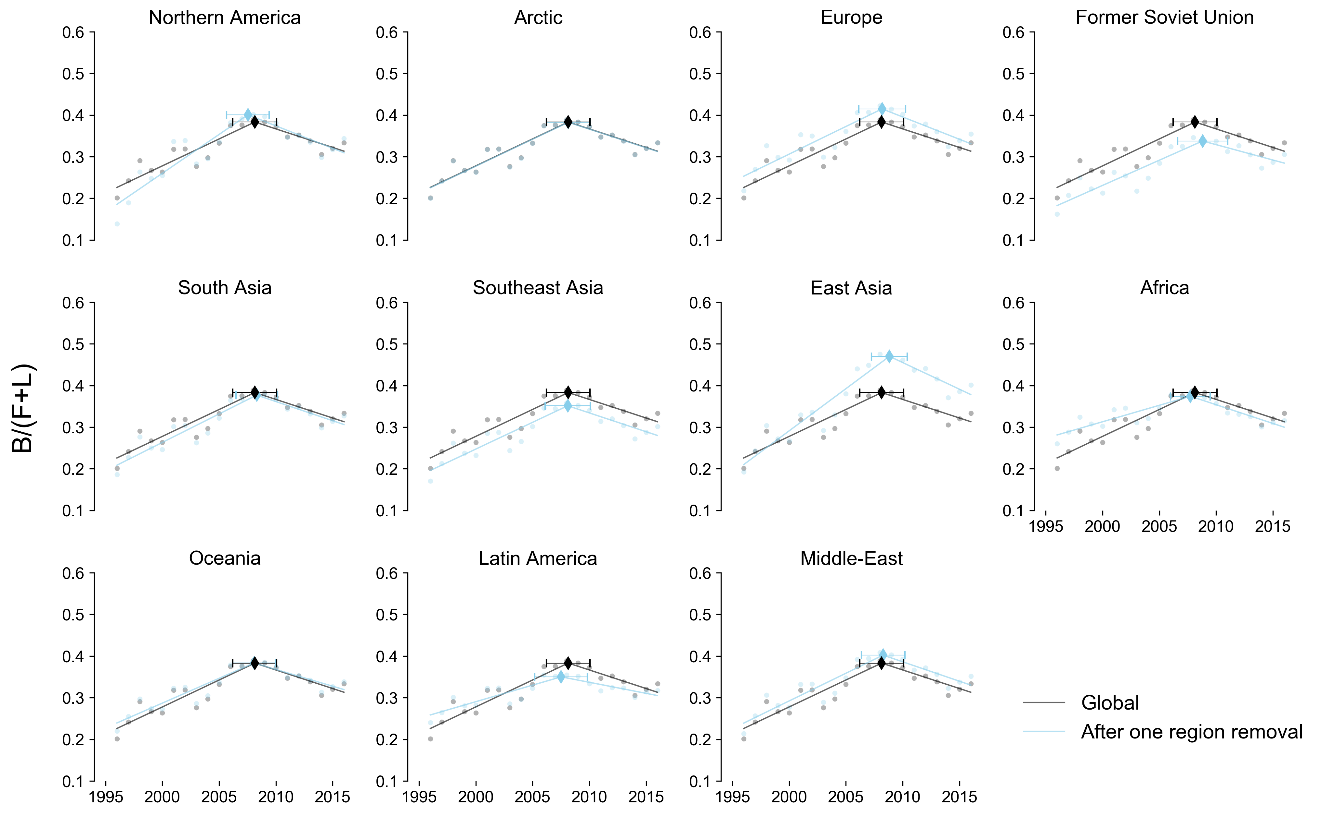


**Figure S5.** Same as Figure S3 but for Jena_s76.


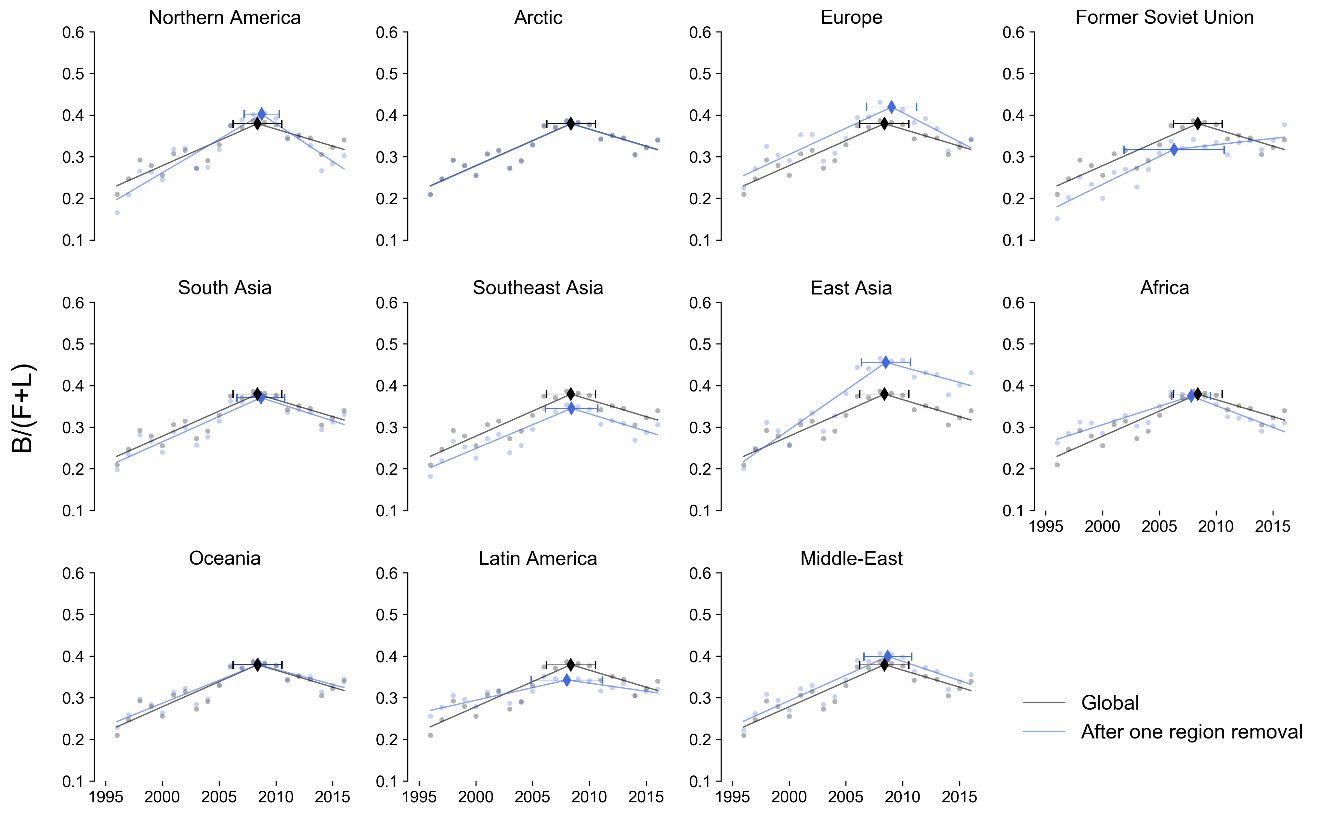


**Figure S6.** Same as Figure S3 but for Jena_s81.


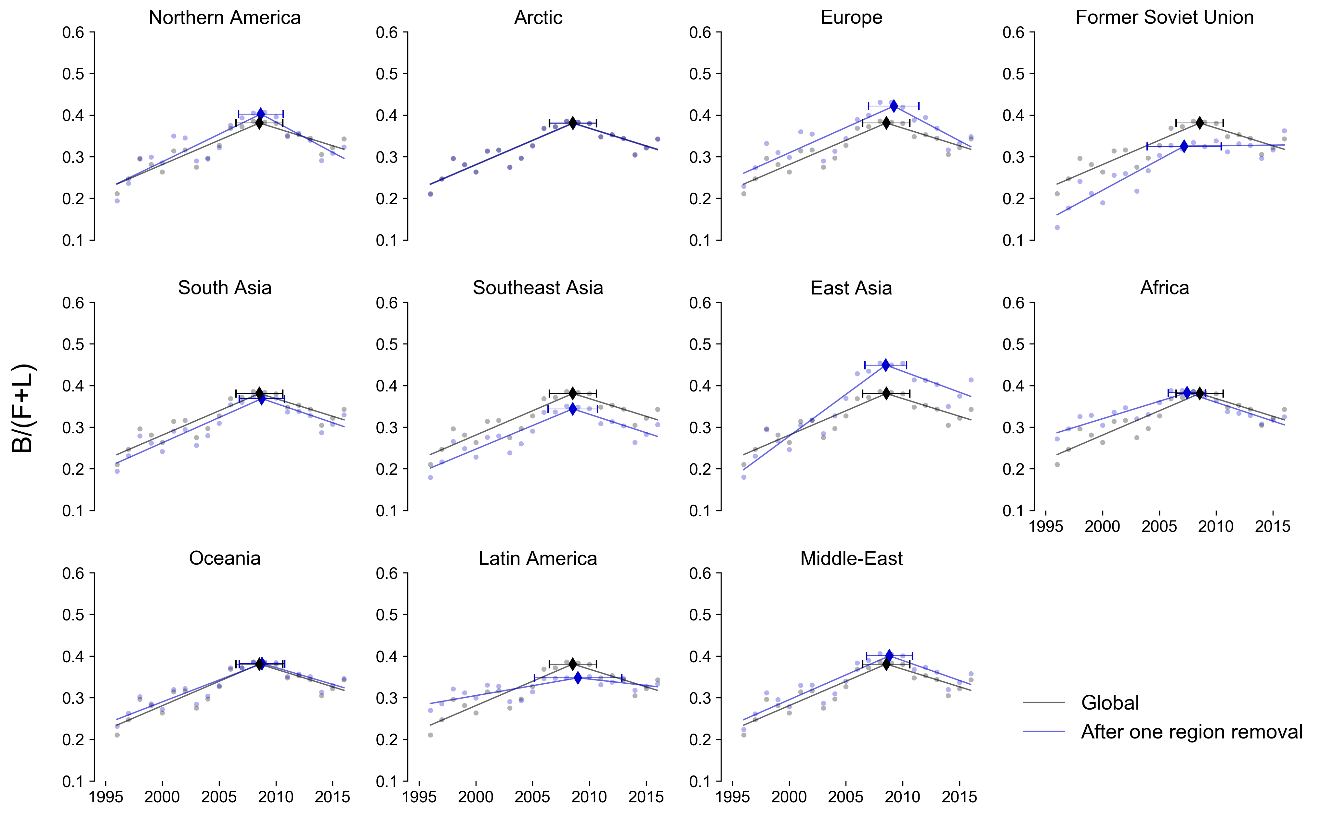


**Figure S7.** Same as Figure S3 but for Jena_s85.


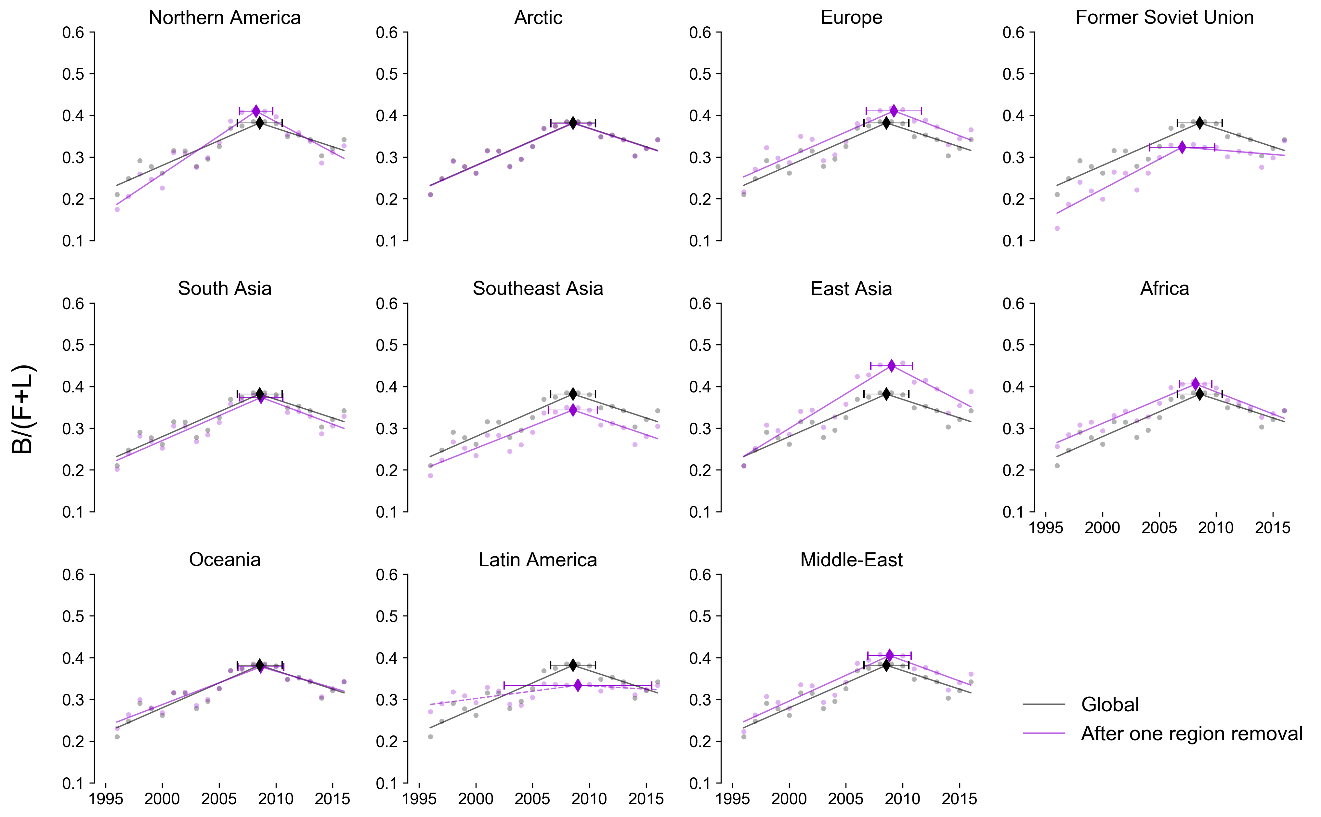


**Figure S8.** Same as Figure S3 but for Jena_s93. The dashed line in the Latin America panel indicates the insignificance of the breakpoint.


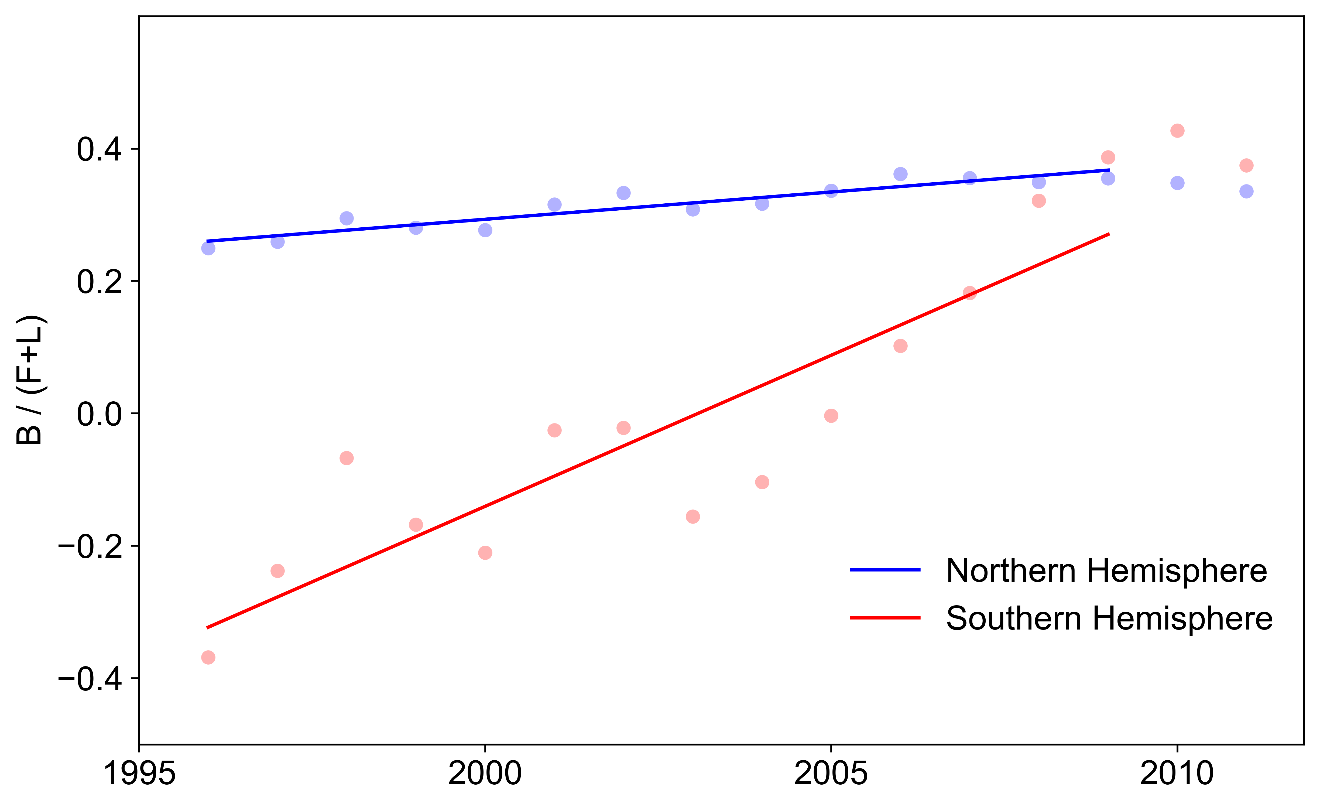


**Figure S9.** Linear regressions of B/(F+L) using B from two-box model based on 5-yr moving averages of carbon fluxes. B in the inversion dataset is calculated by BL−L where L from BLUE is used. Note that two-box model is available until 2013, thus after 5-yr moving average, the analysis period is shown as 1996-2011 instead of 1994-2013 (the original annual values) and the breakpoint can’t be detected due to the short time series after 2009.


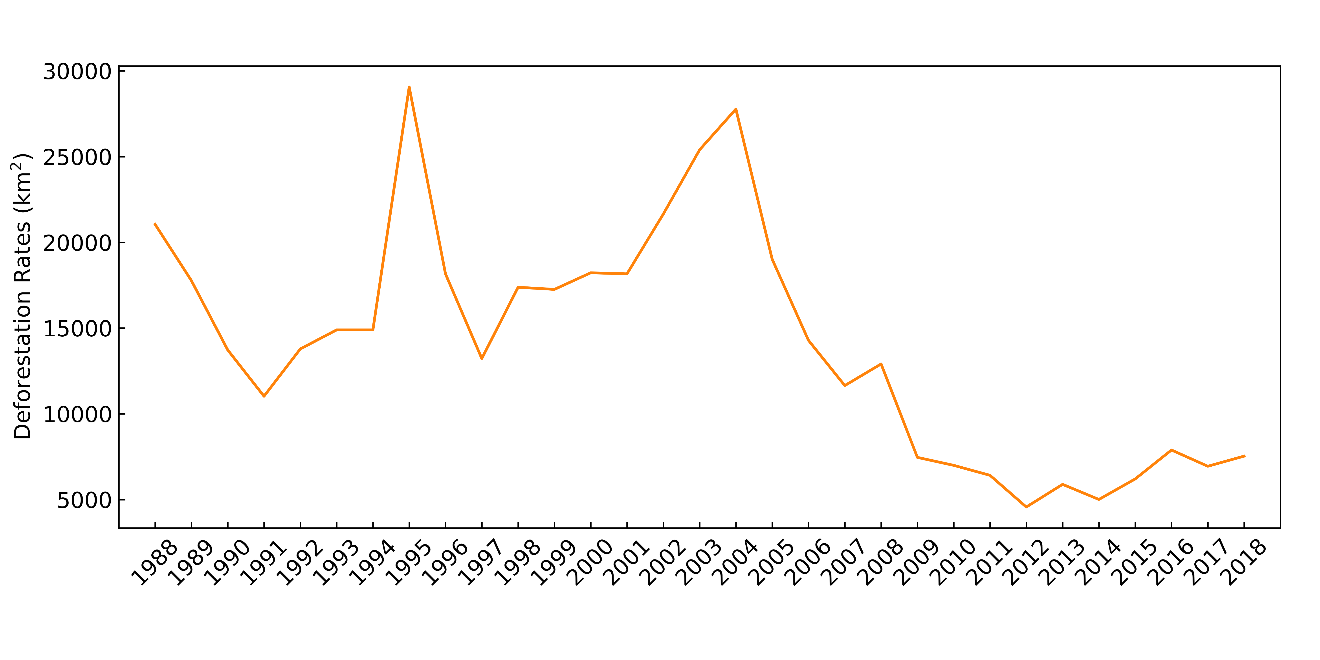


**Figure S10.** Annual deforestation rates in Legal Amazon area (available at http://terrabrasilis.dpi.inpe.br/app/dashboard/deforestation/biomes/legal_amazon/rates). Deforestation rates are calculated based on areas of deforestation greater than 6.25 hectares. Legal Amazon area includes 9 states: Acre, Amapá, Maranhão, Mato Grosso, Pará, Roraima, Tocantins, Rondônia and Amazonas.


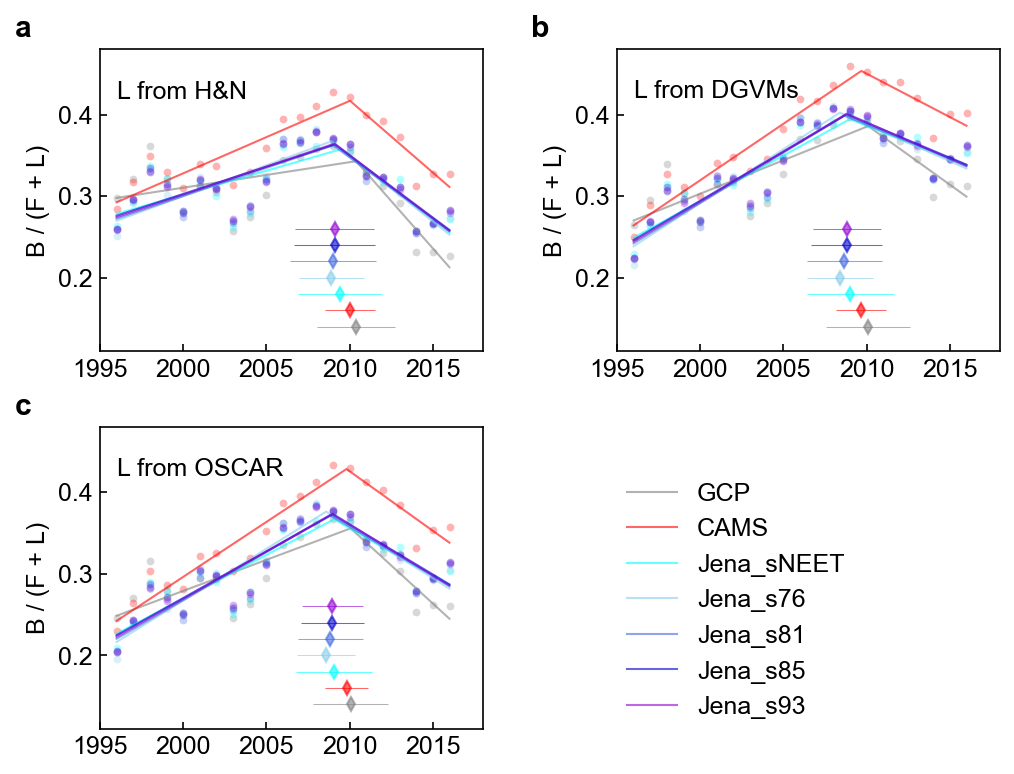


**Figure S11.** Piecewise regressions of B/(F+L) using L from different datasets based on 5-yr moving averages of carbon fluxes to calculate B (BL-L) and B/(F+L) from 1996-2016: (a) L from H&N, (b) L from DGVMs, and (c) L from OSCAR. The dots and lines are the original data and the fitted data respectively. The diamonds and error bars indicate the detected breakpoints with 95% confidence interval. Note that we used 5-yr moving average fluxes, and thus the analysis period is shown as 1996-2016 instead of 1994-2018 (the original annual values).


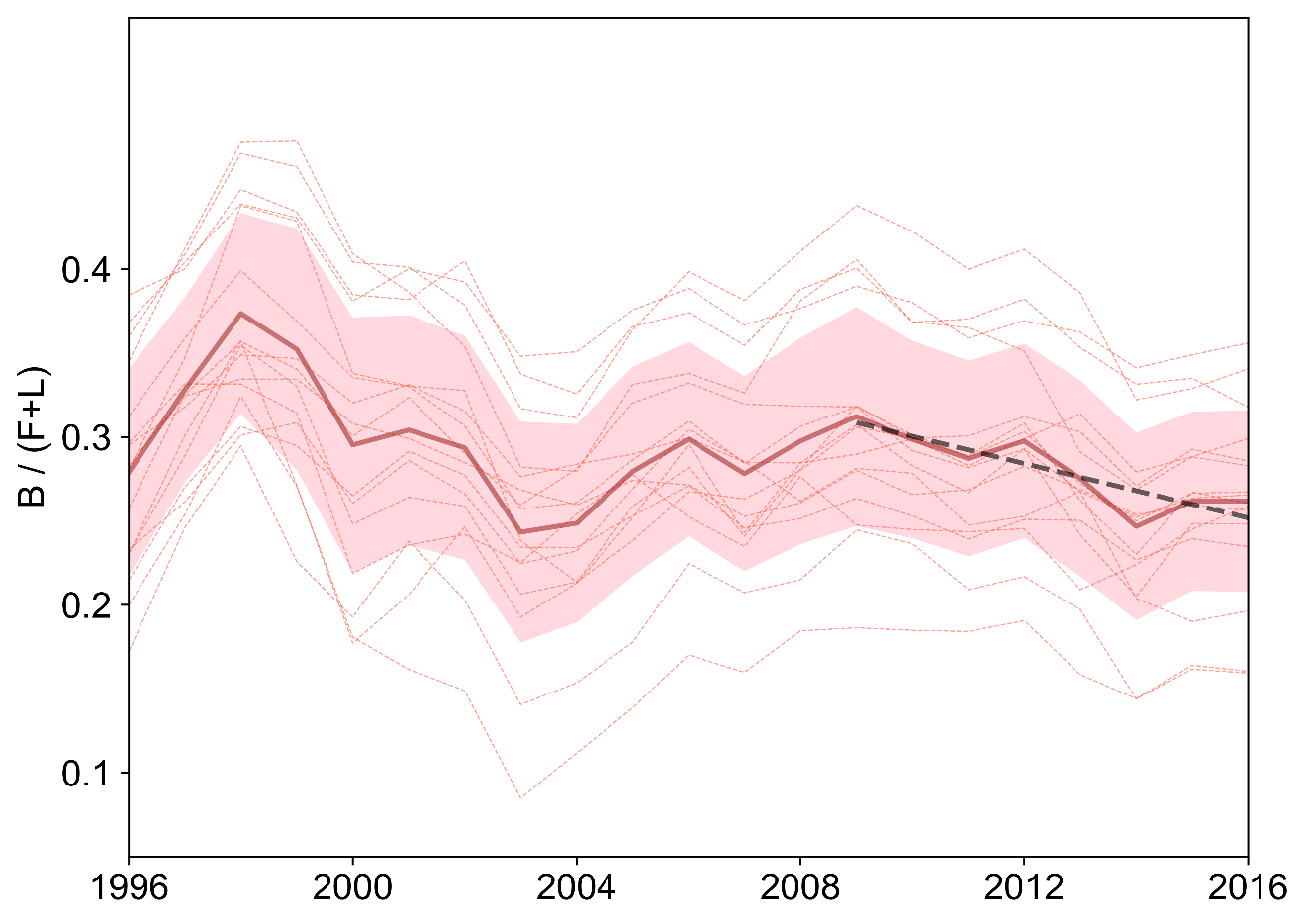


**Figure S12.** B/(F+L) using B from 16 DGVMs models based on 5-yr moving average of carbon fluxes. L from BLUE is used. The dotted lines are B/(F+L) using B from 16 DGVMs models. The solid red line and pink shade are the mean value of 16 models with 1-σ uncertainties. The dashed black line is the linear fitted line during 2009-2016. Note that we used 5-yr moving average fluxes, and thus the analysis period is shown as 1996-2016 instead of 1994-2018 (the original annual values).


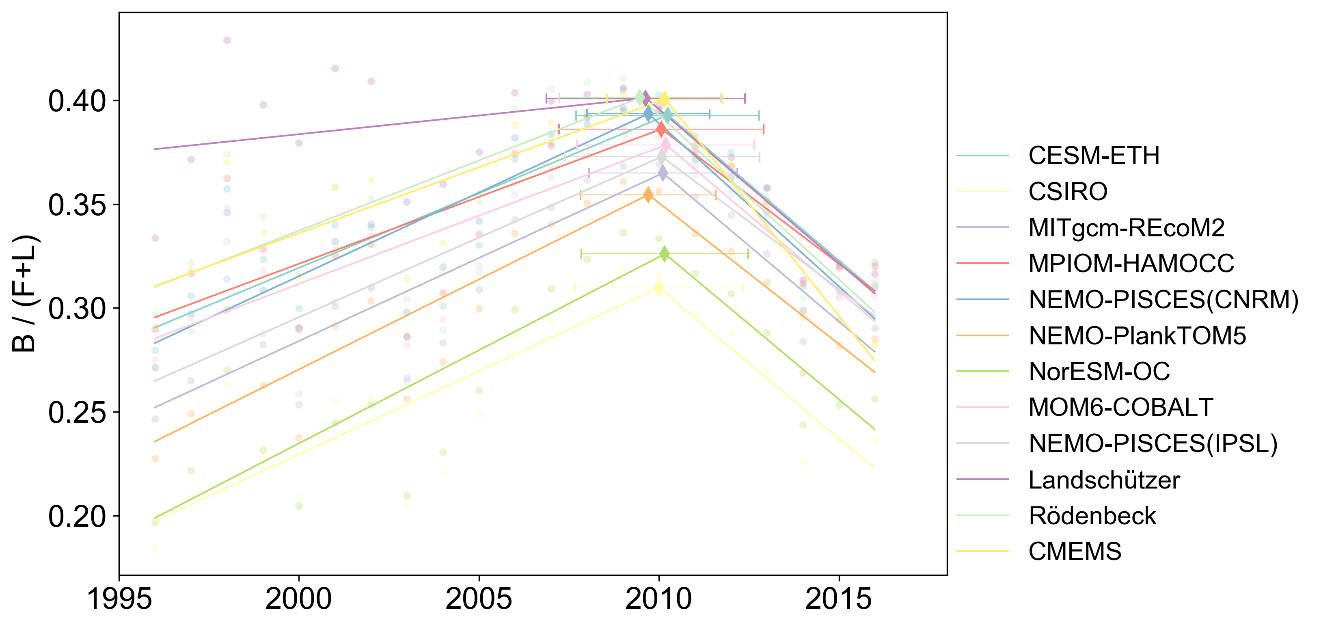


**Figure S13.** B/(F+L) using B calculated as the residue of F, L, global atmospheric CO_2_ growth rate and ocean sink from 9 different ocean models and 3 data-based products in the global carbon project. The dots are the original data. The solid lines are results from piecewise regression. The diamonds and error bars indicate the detected breakpoints with 95% confidence interval. Note that we used 5-yr moving average fluxes, and thus the analysis period is shown as 1996-2016 instead of 1994-2018 (the original annual values).


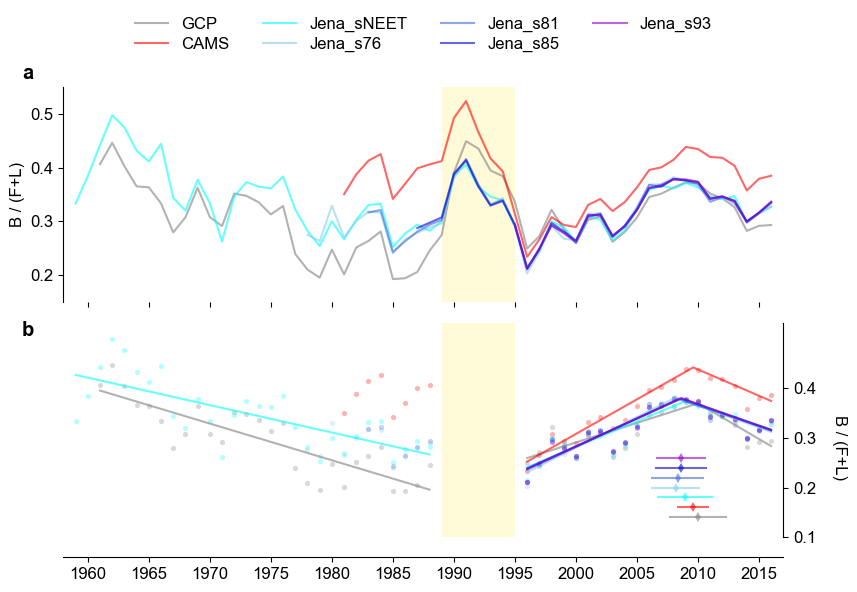


**Figure S14.** Same as Figure 1 but without fossil fuel emission adjustment (see Section 2.1) for Jena data.


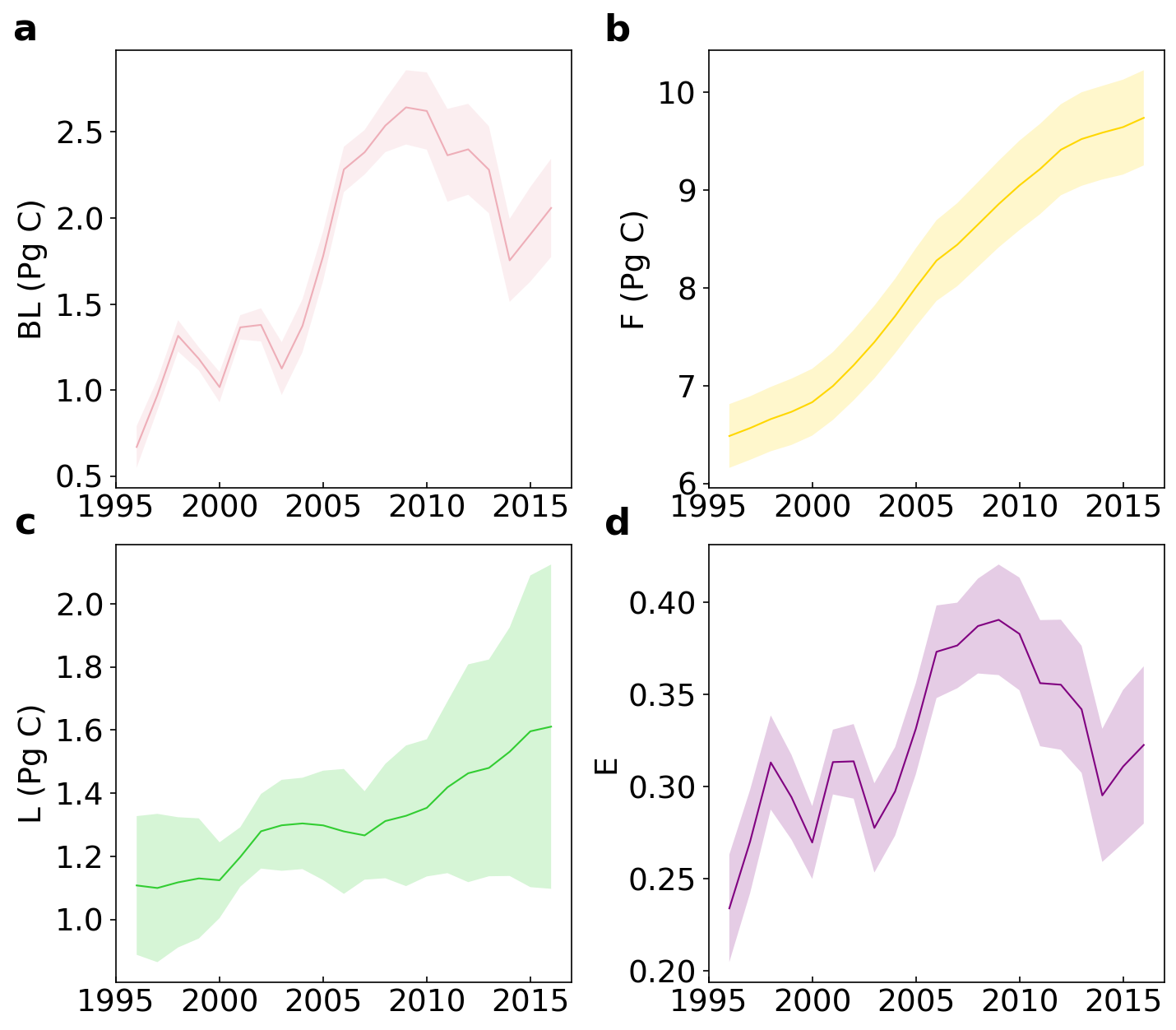


**Figure S15.** Annual carbon fluxes (5-yr moving average) and land sink efficiency (E) after the Pinatubo eruption. Shaded area represents the uncertainty range.


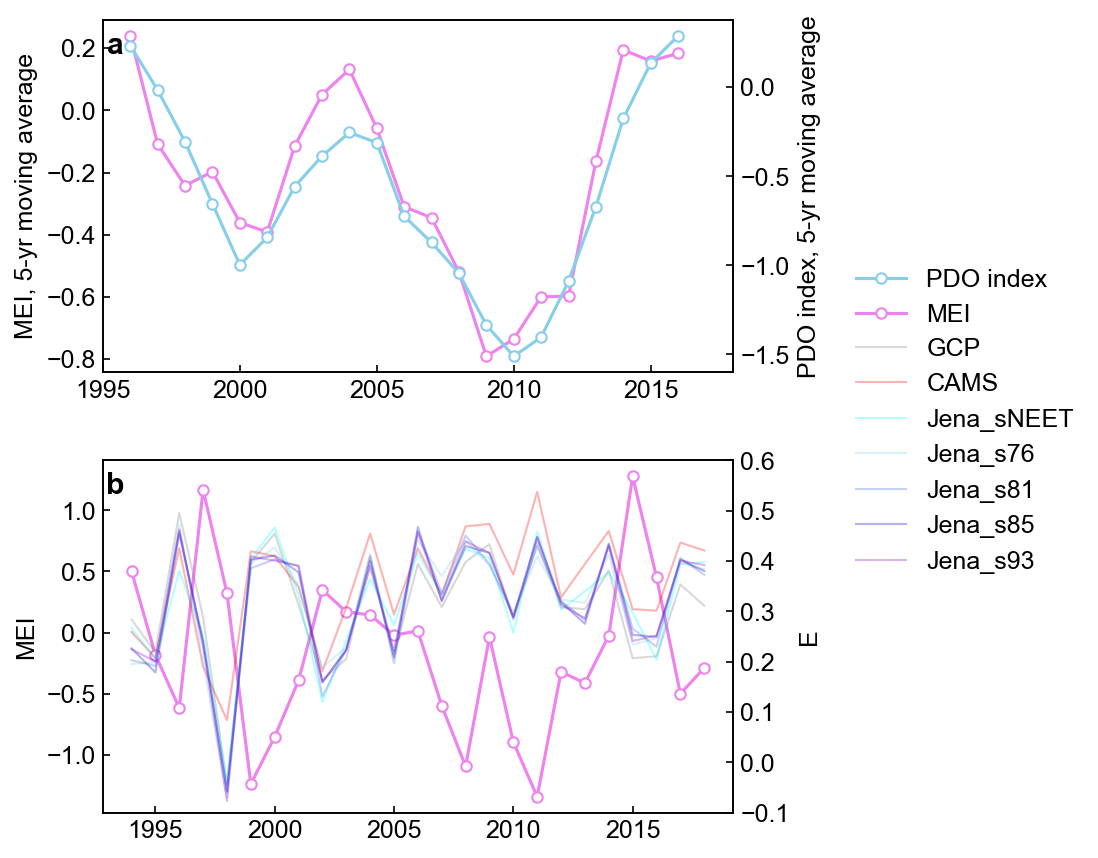


**Figure S16.** MEI and PDO index based on 5-yr moving averages during 1996-2016 (a). Original annual values of MEI and E without 5-yr moving averages during 1994-2018 (b).


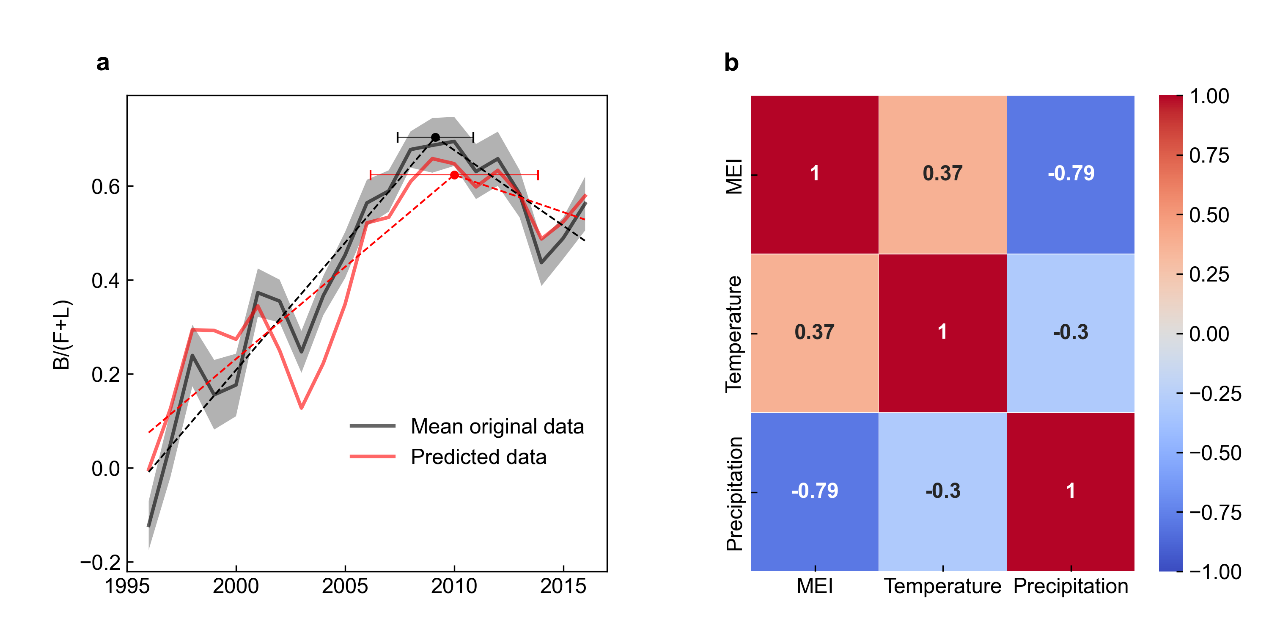


**Figure S17.** Original (black line) and predicted (red line) land sink efficiency based on 5-yr moving average with breakpoint estimations in tropics (a) and Pearson correlation between MEI, tropical temperature and tropical precipitation (b). (a) The black solid line and grey shade are mean value of land sink from CAMS and five Jena datasets divided by F+L with 1-σ uncertainties in tropics based on 5-yr moving average. The predicted land sink comes from multiple linear regression. We use annual tropical temperature, precipitation and global CO_2_ concentration for regression analysis with annual tropical land sink without 5-yr moving average (R^2^=78.3%, p<0.01). Dashed lines represent the piecewise regression result base on 5-yr moving average. The dots and error bars indicate the detected breakpoints with 95% confidence interval. Note that because we find the reversal is dominated by tropical regions, we did the regression analysis only in tropics. The tropical regions include Latin America, Africa and Southeast Asia.


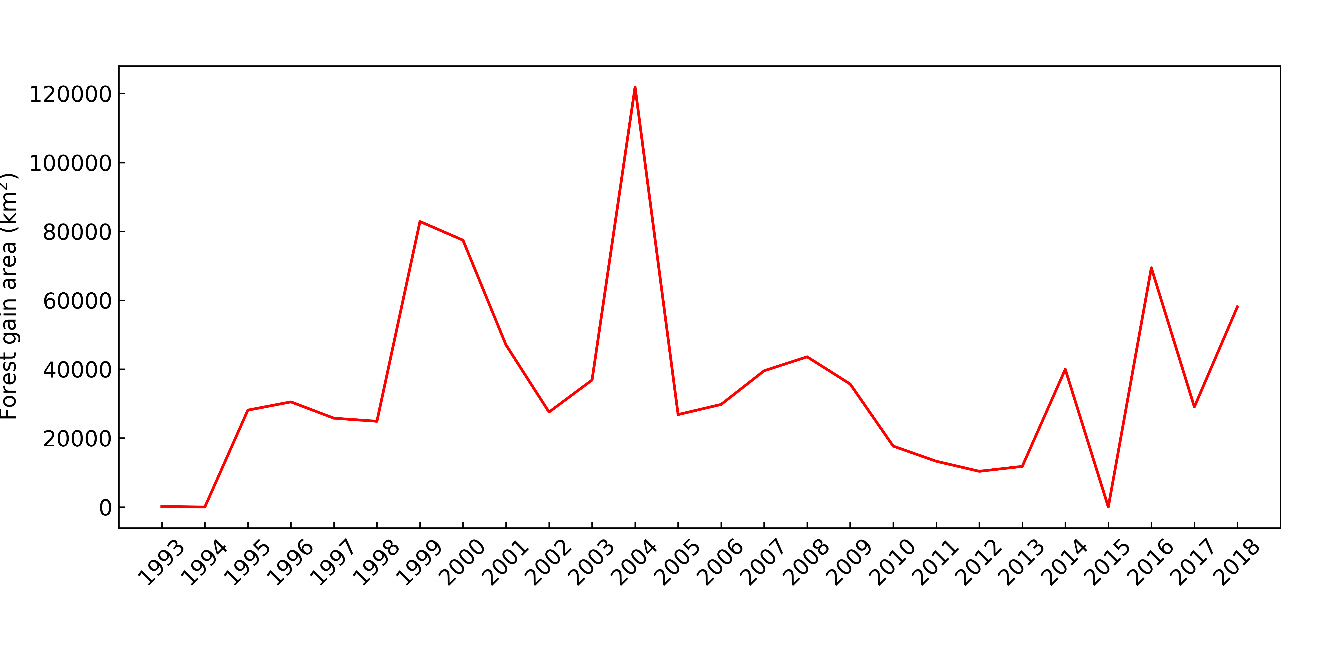


**Figure S18.** Global annual forest area gain from ESA CCI (European Space Agency Climate Change Initiative).

| Flux | Period | Resolution | Data Source |
| --- | --- | --- | --- |
| BL | 1979-2018 | 1.875° latitude × 3.75° longitude | CAMS (<https://apps.ecmwf.int/datasets/data/cams-ghg-inversions/>) [1] |
| BL | 1957-2018 | 3.75° latitude × 5° longitude | Jena_sNEET: Jena CarboScope inversion (http://www.bgc-jena.mpg.de/CarboScope/; run sEXTocNEET_v4.3) [2] |
| BL | 1976-2018 | 3.75° latitude × 5° longitude | Jena_s76: Jena CarboScope inversion run s76oc_v4.3 (http://www.bgcjena.mpg.de/CarboScope/) [3] |
| BL | 1981-2018 | 3.75° latitude × 5° longitude | Jena_s81: Jena CarboScope inversion run s81oc_v4.3 [3] |
| BL | 1985-2018 | 3.75° latitude × 5° longitude | Jena_s85: Jena CarboScope inversion run s85oc_v4.3 [3] |
| BL | 1993-2018 | 3.75° latitude × 5° longitude | Jena_s93: Jena CarboScope inversion run s93oc_v4.3 [3] |
| BL | 1959-2018 | Hemispheric | Two-box model [4] |
| B | 1959-2018 | Global | A residual of F, L, atmospheric CO_2_ growth and ocean sinks from an ensemble of ocean biogeochemical models in the global carbon budget [5] |
| B | 1959-2018 | Global | NBP of the S2 simulation from 16 DGVMs in the global carbon budget [5] |
| F | 1979-2018 | 1.875° latitude × 3.75° longitude | CAMS [1] |
| F | 1959-2018 | Global | Fossil fuel emissions in global carbon budget [5] |
| F | 1957-2018 | 3.75° latitude × 5° longitude | Jena CarboScope inversion [3] |
| L | 1957-2018 | 0.5° latitude × 0.5° longitude | BLUE model [6], |
| L | 1957-2018 | Regional | H&N bookkeeping model (GCP extended the results from 2016 to 2018) [5, 7] |
| L | 1957-2018 | Regional | OSCAR [8] |
| L | 1957-2018 | Global | NBP difference between S2 and S3 simulation from16 DGVMs in the global carbon budget [5] |

**Table S1.** Sources of different datasets used in this study.

|  | 3-yr moving average | | |  | Annual fluxes without moving average | | |  | Replacing 2015 and 2016 with the average of 2014 and 2017, 5-yr moving average | | |
| --- | --- | --- | --- | --- | --- | --- | --- | --- | --- | --- | --- |
|  | Breakpoint | Standard error | P value |  | Breakpoint | Standard error | P value |  | Breakpoint | Standard error | P value |
| GCP | 2010.26 | 2.16 | 0.10 * |  | 2009.00 | 6.64 | 0.80 |  | 2009.73 | 2.36 | 0.24 |
| CAMS | 2010.00 | 1.22 | 0.00 ** |  | 2009.00 | 4.30 | 0.50 |  | 2009.00 | 1.25 | 0.00 ** |
| Jena_sNEET | 2009.26 | 2.30 | 0.08 * |  | 2008.00 | 7.90 | 0.87 |  | 2007.94 | 2.11 | 0.08 * |
| Jena_s76 | 2008.18 | 2.19 | 0.06 * |  | 2008.00 | 5.85 | 0.78 |  | 2007.43 | 1.82 | 0.03 ** |
| Jena_s81 | 2008.68 | 2.18 | 0.08 * |  | 2008.00 | 6.86 | 0.87 |  | 2007.86 | 2.37 | 0.14 |
| Jena_s85 | 2008.98 | 2.10 | 0.08 * |  | 2008.00 | 7.21 | 0.87 |  | 2008.00 | 2.26 | 0.12 |
| Jena_s93 | 2008.99 | 2.22 | 0.10 * |  | 2008.00 | 7.55 | 0.91 |  | 2008.00 | 2.12 | 0.09 * |

**Table S2.** Sensitivity tests on the breakpoint detection. Notes: *, ** represent the significance at the 0.1 and 0.05 levels, respectively.

|  | Standard deviation | Interannual variability |
| --- | --- | --- |
| BL (Pg C) | 0.169 | 0.604 |
| F (Pg C) | 0.407 | 1.139 |
| L (Pg C) | 0.236 | 0.151 |
| E | 0.028 | 0.043 |

**Table S3.** The uncertainty and interannual variability of E during 1996-2016 after 5-yr moving average.

**References:**

1 Chevallier, F., et al. (2010), CO_2_ surface fluxes at grid point scale estimated from a global 21 year reanalysis of atmospheric measurements, *Journal of Geophysical Research*, *115*(D21).

2 Rödenbeck, C., S. Zaehle, R. Keeling, and M. Heimann (2018), How does the terrestrial carbon exchange respond to inter-annual climatic variations? A quantification based on atmospheric CO_2_ data, *Biogeosciences*, *15*(8), 2481-2498.

3 Rödenbeck, C., Houweling, S., Gloor, M., & Heimann, M (2003), CO_2_ flux history 1982-2001 inferred from atmospheric data using a global inversion of atmospheric transport., *Atmospheric Chemistry and Physics*, *3, 1919-1964*.

4 Ciais, P., et al. (2019), Five decades of northern land carbon uptake revealed by the interhemispheric CO_2_ gradient, *Nature*, *568*(7751), 221-225.

5 Friedlingstein, P., et al. (2019), Supplemental data of Global Carbon Budget 2019 (Version 1.0) [Data set], *Global Carbon Project*.

6 Hansis, E., S. J. Davis, and J. Pongratz (2015), Relevance of methodological choices for accounting of land use change carbon fluxes, *Global Biogeochemical Cycles*, *29*(8), 1230-1246.

7 Houghton, R. A., and A. A. Nassikas (2017), Global and regional fluxes of carbon from land use and land cover change 1850-2015, *Global Biogeochemical Cycles*, *31*(3), 456-472.

8 Gasser, T., L. Crepin, Y. Quilcaille, R. A. Houghton, P. Ciais, and M. Obersteiner (2020), Historical CO_2_ emissions from land-use and land-cover change and their uncertainty, *Biogeosciences Discuss*, *2020*, 1-43.
